# Effect of fresh-frozen plasma transfusion on intraventricular hemorrhage risk in preterm infants with prolonged APTT: a target trial emulation with causal forest analysis

**DOI:** 10.3389/fnins.2026.1950034

**Published:** 2026-09-07

**Authors:** Bingyi Tang, Qinyi Yu, Jialin Zhang, Xinyu Duan, Teng Meng, Zhengdong Chen, Qingqi Ran, Yi Zhang, Siqi Tian, Zhangxue Hu, Wenjie Peng

**Affiliations:** 1 Department of Pediatrics, Daping Hospital, Army Medical University, Chongqing, China; 2 Department of Breast and Thyroid Surgery, Daping Hospital, Army Medical University, Chongqing, China; 3 Operation Center of Huashan Hospital, Fudan University, Shanghai, China

**Keywords:** fresh-frozen plasma, intraventricular hemorrhage, preterm infants, prolonged activated partial thromboplastin time, target trial emulation

## Abstract

**Background:**

Coagulopathy is a common risk factor in the occurrence of intraventricular hemorrhage (IVH) and subsequent poor neurological outcomes in preterm infants. The causal effect of fresh-frozen plasma (FFP) transfusion and IVH needed to be identified urgently.

**Methods:**

Within the framework of target trial emulation (TTE), we mimic the process of a randomized controlled trial based on retrospective neonatal clinical data collected at Daping Hospital from 2015 to 2025, ultimately including 748 preterm infants with gestational age ≤34 weeks and prolonged activated partial thromboplastin time ≥70 s. Multiple causal inference models were conducted to minimize confounding and covariate imbalance, including inverse probability weighting with generalized boosted models (GBM-IPW) method, GBM-doubly robust (GBM-DR) estimation, covariate balancing propensity score (CBPS) model and other sensitivity analyses. Causal forests algorithm further triangulated the main results and explore individual-level heterogeneity and key determinants about FFP.

**Results:**

All causal models consistently demonstrated that FFP transfusion exerted no overall estimated protective effect against IVH (all odds ratio (OR) ≈ 1; *p >* 0.4). GA was found a protective factor of IVH and modified the effect of FFP transfusion (*p* < 0.05). According the cutoff of 32.3-week cutoff, FFP transfusion significantly increased IVH risk in the more premature subgroup (OR = 2.16, *p* = 0.02). Causal forests also found individual heterogeneity exists between FFP and IVH, and relevant high-risk subgroups about FFP transfusion can be stratified by prematurity, Apgar scores, and coagulopathy.

**Conclusion:**

Routine FFP transfusion is not recommended for IVH prevention in preterm infants. FFP significantly increases IVH risk in more premature infants, while the adverse association of FFP attenuated with increasing GA.

## 1 Introduction

Intraventricular hemorrhage (IVH) is a prevalent and severe hemorrhagic condition, especially causing morbidity and long-term neurodevelopmental outcomes in preterm neonates (Argyropoulou et al., 2022). Multiple factors, such as the intrinsic fragility of the germinal matrix vasculature or systemic hemodynamic instability, influence the occurrence of IVH, whose incidence across all grades increased significantly with lower gestational age (GA) and birth weight (BW). IVH affects over 20% of infants born before 32 weeks. Although severe IVH is rare in moderate-to-late preterm infants, this subgroup represents 84% of all preterm infants, suggesting an undeniable disease burden (Argyropoulou et al., 2022; Rees et al., 2025). Furthermore, microbleeds can potentially trigger white matter injury and higher risk of long-term neurological impairments, including cerebral palsy, neurodevelopmental delay, epilepsy, and so on (Egesa et al., 2021). Current numerous medical measures such as antenatal corticosteroids, respiratory support, and fresh-frozen plasma (FFP) transfusion lack evidence of efficacy in preventing IVH (Egesa et al., 2021; Guzzardo and Regling, 2022). Therefore, precise preventive strategies about IVH are critically needed in the management of preterm infants.

Coagulopathy has been recognized as a central, modifiable intrinsic risk factor for IVH in preterm infants (Dani et al., 2009; Houben et al., 2020). The levels of most coagulation factors approximate only 50% of adult reference values during the perinatal period, which could be markedly more severe in preterm infants (Dani et al., 2009; Zerra and Josephson, 2021). Evidence suggests that FFP transfusion can correct approximately 40–60% of the prolonged clotting values (Roberts et al., 2022) and is frequently administered for correcting coagulopathy—commonly defined as prolonged activated partial thromboplastin time (APTT) (~1.5 × upper limit of normal in neonates)—yet whether this practice reduces IVH remains unexamined across heterogeneous risk strata is controversial (Neary et al., 2013). However, the association of prolonged APTT or prothrombin time (PT) and IVH risk remain inconclusive (Neary et al., 2015; Jiang et al., 2025; Natarajan and Shankaran, 2016). Guidelines recommend FFP transfusion primarily for neonates with active hemorrhage or coagulopathy before risky operations (Christensen et al., 2014a). Preventive use of FFP in neonates remain controversial, owing to deficient neonatal transfusion standards (Christensen et al., 2014a). Routine prophylactic FFP administration does not confer mortality benefit in preterm infants and may raises various adverse events (Adeshina et al., 2026; Wang et al., 2025). Preterm infants with severe sepsis, extremely low birth weight (<1,000 g), or prolonged mechanical ventilation–are more likely to receive FFP and inherently carry elevated baseline IVH risk (Maruyama et al., 2012). Uniform treatment ignores divergent effects of FFP determined by baseline coagulation, platelet level and GA. Methodological breakthroughs are thus urgently required to enrich clinical evidence under ethical constraints.

According the substantial ethical limitation to conducting pragmatic RCTs in vulnerable preterm neonates (Maruyama et al., 2012), TTE provides a structured framework for causal assessment using real-world data and effectively minimizes confounding bias (Dogra et al., 2020). Machine learning is widely used to capture complex, nonlinear interactions among a range of clinical variables (Maruyama et al., 2012; Saeedi et al., 2025; Lee et al., 2021). Furthermore, causal forest model was an emerging method for precise risk prediction and heterogeneous effect evaluation of FFP (Natarajan and Shankaran, 2016).

Through target trial emulation (TTE) and causal forest analysis, preterm infants born at ≤34 weeks’ gestation, presenting with an prolonged APTT and no initial IVH, were finally included. Then this population-based study aim to estimate the average causal effect of prophylactic FFP transfusion on IVH risk and provide a personalized risk–benefit framework to advance the prevention of IVH.

## 2 Methods

### 2.1 Ethics statement

The study fully comply with the Strengthening the Reporting of Observational Studies in Epidemiology (STROBE) statement and the principles of the Declaration of Helsinki (Saeedi et al., 2025), approved by the Medical Ethics Committee at Daping Hospital (approval no.: 2026.152) and registered in the Chinese Clinical Trial Registry (registration no.: ChiCTR2600125060) on 2026-05-20. All analyses were conducted using identified, routinely collected retrospective clinical data, accordingly, the requirement for informed consent was waived by the institutional review board.

### 2.2 Study design

Based on the retrospective real-world data, we designed the TTE procedure to emulate a parallel-group RCT. The TTE framework was strictly predefined according to standardized causal inference principles with six core components: (1) eligibility criteria: neonates with a GA ≤ 34 weeks and prolonged APTT (≥70 s), with eligibility assessed at time zero; (2) treatment strategies: initiation of a qualifying FFP transfusion within 24 h after time zero versus no FFP transfusion during the treatment-assessment window or subsequent follow-up; (3) assignment mechanism: treatment assignment begin during the prespecified 24-h grace period after time zero, with baseline confounding addressed using propensity-score (PS) weighting; (4) outcome definition: all-grade IVH’s occurrence within 28 days after time zero; (5) follow-up period: from time zero until the first diagnosis of IVH, with infants without IVH followed until death, discharge, transfer, or 28 days after time zero, whichever occurred first; (6) causal estimands: average treatment effect (ATE). Effect sizes were further presented as odds ratios (OR) and 95% confidence intervals (CI) (Dogra et al., 2020). Identification of the causal effects of FFP on IVH relied on the assumptions of consistency, conditional exchangeability, and positivity. Additional *post hoc* 24-h landmark sensitivity analysis was conducted to address potential immortal time bias or selection bias.

We mainly archived the TTE framework relied on the generalized boosted modeling-based inverse probability weighting (GBM-IPW) approach, which minimized baseline confounding and emulated the group assignment in randomized studies. Propensity score (PS) estimation via GBM accommodates nonlinear relationships and high-order interactions present in baseline covariates. Weighted statistical analyses yielded causal effect estimates consistent with outcomes of properly implemented RCT (Dogra et al., 2020). Covariate balance in weighted groups was assessed using standardized mean difference (SMD), with SMD < 0.10 defined as adequate balance. Several sensitivity tests, such as weight trimming and modified CBPS strategies, were carried out to confirm result robustness. Causal forest was further adopted to assess varying treatment effects across different subgroups (Lee et al., 2021).

### 2.3 Data description

**Figure 1:**
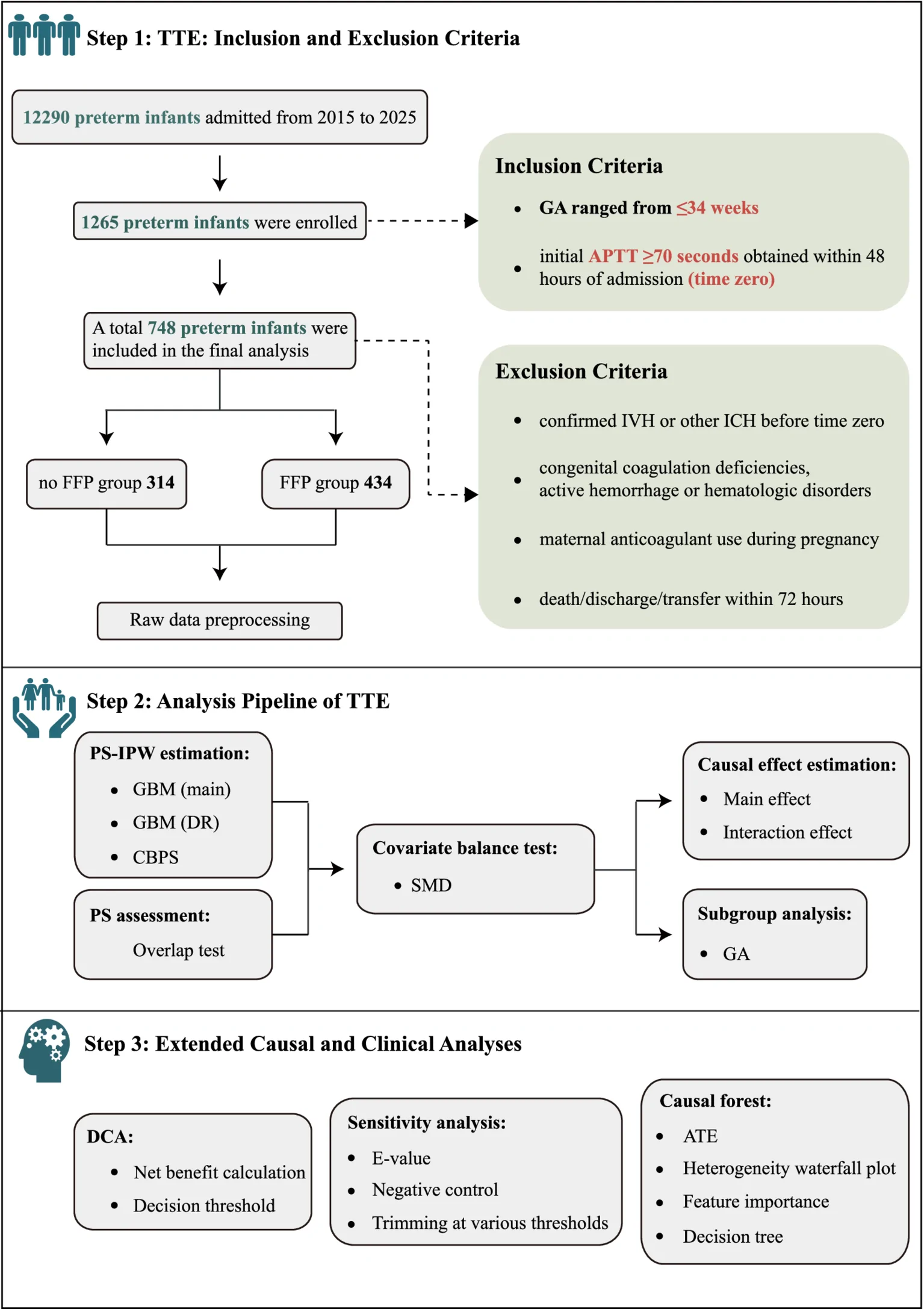
Flowchart of the research project. APTT, activated partial thromboplastin time; ATE, average treatment effect; CBPS, covariate balancing propensity score; DCA, decision curve analysis; FFP, fresh frozen plasma; GA, gestational age; GBM, generalized boosted model; GBM (DR), generalized boosted model (doubly robust); IVH, intraventricular hemorrhage; PS-IPW, propensity score-inverse probability weighting; SMD, standardized mean difference; TTE, target trial emulation.

Clinical data from preterm infants admitted to the Neonatal Intensive Care Unit (NICU) at Daping Hospital in Chongqing between January 2015 and December 2025. Eligible infants were born at ≤34 weeks of gestation, admitted within 24 h after birth, and had an initial APTT ≥70 s measured within 48 h of admission, which was defined as time zero. The published neonatal reference suggested considering intervened approach in which APTT >74 s on postnatal day 5 (Christensen et al., 2014b). We lowered the threshold to 70s in align with local validated neonatal reference intervals, an APTT ≥70 s is used define coagulation dysfunction in this study (Christensen et al., 2014b). Cranial ultrasonography performed between hospital admission and the 24-h landmark confirmed the absence of IVH before the start of landmark follow-up. Exclusion criteria included pre-existing IVH or other intracranial hemorrhage, congenital coagulation deficiencies or hematologic disorders, maternal anticoagulant use during pregnancy, and loss to follow-up for the death, discharge or transfer within 72 h after birth. Based on the administration of a single FFP transfusion (10–15 mL/kg.h) (Adeshina et al., 2026) within 24 h after time zero, all subjects were classified into the treatment group and control group. The control group did not receive the FFP transfusion in the subsequent follow-up period. Finally, 748 eligible preterm infants were ultimately enrolled from 12,290 preterm infants, comprising 434 cases in the treatment group and 314 cases in the control group (Figure 1).

The incidence of any-grade of IVH within 28 days after time zero was defined as the main outcome. In addition, the outcome was diagnosed through a series of routine cranial ultrasound screening and graded according to the Papile classification (I-IV) (Adeshina et al., 2026). Moreover, the period between time zero and the first diagnosis of IVH, hospital discharge, transfer, death or 28 days after time zero was defined as follow-up time. Covariates collected for adjustment and analysis including demographic characteristics, perinatal factors, postnatal status, comorbidities and other therapeutic measures (Maruyama et al., 2012; Dogra et al., 2020; Natarajan and Shankaran, 2016). All collected covariates were measured before or at the time of treatment assignment to avoid post-treatment bias and directly retrieved from admission records and laboratory databases. When addressing missing raw data, we used mean imputation for continuous variables with <5% missingness and maximum likelihood imputation for categorical variables, thereby minimizing potential bias (Lee et al., 2011).

### 2.4 Statistic analysis

#### 2.4.1 Comparison of baseline data statistics

Continuous variables were presented as mean ± standard deviation (SD) or median with interquartile range (IQR), as appropriate. Categorical variables were expressed as frequencies and percentages. Differences in baseline characteristics between the two groups were compared using the Student*’*s *t*-test or the Mann-Whitney *U* test for continuous variables and the Chi-square test or Fisher*’*s exact test for categorical variables (Crump et al., 2009).

#### 2.4.2 Inverse probability weighting based on generalized boosted model

To mitigate confounding bias from observed covariates, covariate balance before and after GBM-IPW weighting was evaluated with standardized mean difference (SMD). And the absolute SMD of each variable <0.1 was considered adequate (Crump et al., 2009). Moreover, we trimmed the upper and lower 2.5% of the stabilized weight to reduce sensitivity of extreme weights, subsequently re-normalizing weights (Zhao et al., 2023). After identified covariates balance, we implemented a weighted logistic regression model to estimate the odds ratio (OR) of IVH associated with FFP transfusion, along with its 95% confidence interval (CI) (Zhao et al., 2023; Lai et al., 2026).

#### 2.4.3 Supplementary analysis

Two supplementary sensitivity analyses were implemented to evaluate result stability and adjust for residual confounding: (1) GBM-based doubly robust (GBM-DR) estimation incorporated additional adjustment for GA within weighted logistic regression, enhancing result consistency against potential model misspecification (Shardell et al., 2024; Choi et al., 2020); (2) covariate balancing propensity score (CBPS) served as an alternative weighting strategy. It jointly refines PS and covariate balance, and corroborates the primary GBM-IPW findings (Li and Li, 2021).

#### 2.4.4 Effect modification of GA

To examine effect modification of GA, an interaction term between FFP exposure and GA incorporated into a GBM-IPW logistic regression model was conducted. GA was mean-centered prior to model fitting to alleviate multicollinearity (McCabe et al., 2022). A primary IPW model included the interaction term (FFP × GA-centered), while a secondary, fully adjusted model additionally controlled for birth weight and sex, both established clinical confounders of IVH risk. A two-sided *p* < 0.05 for the interaction term indicated meaningful effect modification. Subsequently, the delta method was applied to estimate the inflection point of GA and its 95% CI. The formula of this model was specified as follows:

$$\begin{array}{c} \log[\frac{P(Y=1|X)}{1-P(Y=1|X)}]=\beta_{0}+\beta_{1}\cdot \mathrm{FFP}+\beta_{2}\cdot (\mathrm{GA}-\bar{\mathrm{GA}})\\ +\beta_{3}\cdot \mathrm{FFP}\cdot (\mathrm{GA}-\bar{\mathrm{GA}}) \end{array}$$

where *Y* denotes IVH occurrence (1 = yes, 0 = no), and GĀ represents the overall sample mean GA. The interaction coefficient *β₃* quantifies the rate of change in the log-odds ratio per additional day of gestation. The marginal predicted probability difference were calculated to quantify the continuous moderating effect of GA (Šinkovec et al., 2019; Jamian et al., 2025). Following confirmation of a statistically significant interaction, the cohort was dichotomized at the critical point of GA into low- and high-GA subgroups, and aOR for IVH were estimated separately for each subgroup and the overall cohort.

#### 2.4.5 Decision curve analysis

The individualized predicted probability of IVH were estimated for each neonate using the GBM-DR model incorporating the FFP-GA interaction. Based on this, decision curve analysis (DCA) evaluated the net clinical benefit within a clinically informative threshold probability range of 0 to 50% (at 1% increments) (Suhas et al., 2023; VanderWeele and Ding, 2017).

#### 2.4.6 E-value analysis

E-value sensitivity analysis was performed to assess the robustness of results against unmeasured confounding, whose formulas proposed by VanderWeele and Ding:for an OR ≥1, $E=\text{OR}+\sqrt{\text{OR}\times (\text{OR}-1)}$

for OR <1, $E=\frac{1}{\text{OR}}+\sqrt{\frac{1}{\text{OR}}\times (\frac{1}{\text{OR}}-1)}$

Two E-values were computed based on the point estimate of OR and the limit of 95% CI closest to the null value (OR = 1), representing the minimum strength of association required for an unmeasured confounder to fully explain the observed association or to render it statistically non-significant (Blank et al., 2025; Haneuse et al., 2019).

#### 2.4.7 Negative control analysis

A negative control analysis was conducted using birth weight (BW) to examine the potential for residual confounding, which could not be affected by FFP transfusion (Iacobucci et al., 2016). The average treatment effect of FFP on BW was estimated using IPW-adjusted linear regression models, including an initial unadjusted model and a GA-adjusted model. A statistically significant association (two-sided *p* < 0.05) in either model would challenge the assumption of adequate covariates balance and indicate the presence of residual confounding.

#### 2.4.8 Global average treatment effect and heterogeneity

Combined with a causal forest algorithm, the global average treatment effect (ATE) was calculated to evaluate the overall impact of FFP transfusion on IVH risk. Results were reported as risk differences with corresponding 95% CI and *p*-values (Haneuse et al., 2019; Lipsitch et al., 2010). Through the *grf* package in R, we further explore this heterogeneous treatment effect (HTE) by estimateing the conditional average treatment effect (CATE) for each individual, namely the individual treatment effect (ITE), whose mathematical formulation below:

τ(x)=E[Y(1)−Y(0)∣X=x]

where *Y* represents IVH, the *Y*(1) and *Y*(0) are the potential outcomes under treatment and control, respectively, and *X* are the matrix of 16 baseline covariates. The model was trained with 3,000 trees to ensure algorithmic stability of the estimates (Lipsitch et al., 2010).

#### 2.4.9 Patient stratification and interpretability

Based on the point estimates and variance of the ITEs, 95% CI was constructed for each patient. Patients were subsequently stratified into three response phenotypes: beneficial (upper bound of 95% CI < 0), harmful (lower bound of 95% CI > 0), and neutral (95% CI crossing 0). This distribution was visualized using a waterfall plot.

Variable importance scores were extracted from the causal forest model to explore the baseline factors driving this heterogeneity in treatment response. Finally, a classification and regression tree (CART) was fitted using the *rpart* package to identify relevant subgroups and convert the complex causal forest outputs into interpretable clinical rules. The tree model used the estimated ITEs as the target variable and baseline covariates as predictors, sequentially partitioning the cohort to identify specific high-risk or highly responsive clinical subgroups (Haneuse et al., 2019).

### 2.5 Landmark analysis

To lower the risk of potential immortal time bias arising from the 24-h treatment-assessment window, we conducted a *post hoc* landmark analysis at 24 h after time zero, consistent with recent methodological recommendations (Duchesneau et al., 2022). We included those patients who were alive, remained hospitalized, and were free of diagnosed IVH at the landmark window. Treatment status was determined according to FFP administration during the 24-h exposure-assessment window, and follow-up for both groups began at the same landmark time. On the other hand, the primary outcome of IVH’s incidence was the sole event of interest. Follow-up ended at IVH diagnosis, whereas infants without IVH were censored at death, discharge, transfer, or 28 days after time zero, whichever occurred first. Logistic PS weighting was repeated in the landmark cohort using covariates measured before or at time zero to estimate the ATE without weight trimming (Austin and Stuart, 2015). Balance was considered adequate when all absolute SMDs were <0.10. Weighted logistic and Cox proportional hazards models with robust sandwich standard errors were used to estimate the 28-day OR and landmark-based HR for IVH, respectively.

## 3 Results

A total of 748 preterm infants with a GA ≤ 34 weeks (238 days) and an initial APTT ≥70 s were included in the final analysis, including 434 cases in the FFP transfusion group and 314 cases in the non-transfusion group (Figure 1). Among the population, any-grade IVH within 28 days occurred in 146 infants after treatment assignment. More specifically, 95 (21.9%) developed IVH in treatment group (*n* = 434) and 51 (16.2%) in control group (*n* = 314), with no statistical significance (*p* = 0.067). In addition, all the missing data of covariates was less than 5%, whose missing rates were summarized in Supplementary Table 1. Missing continuous variables were imputed using mean imputation and categorical variables were imputed using maximum likelihood estimation.

**Figure 2:**
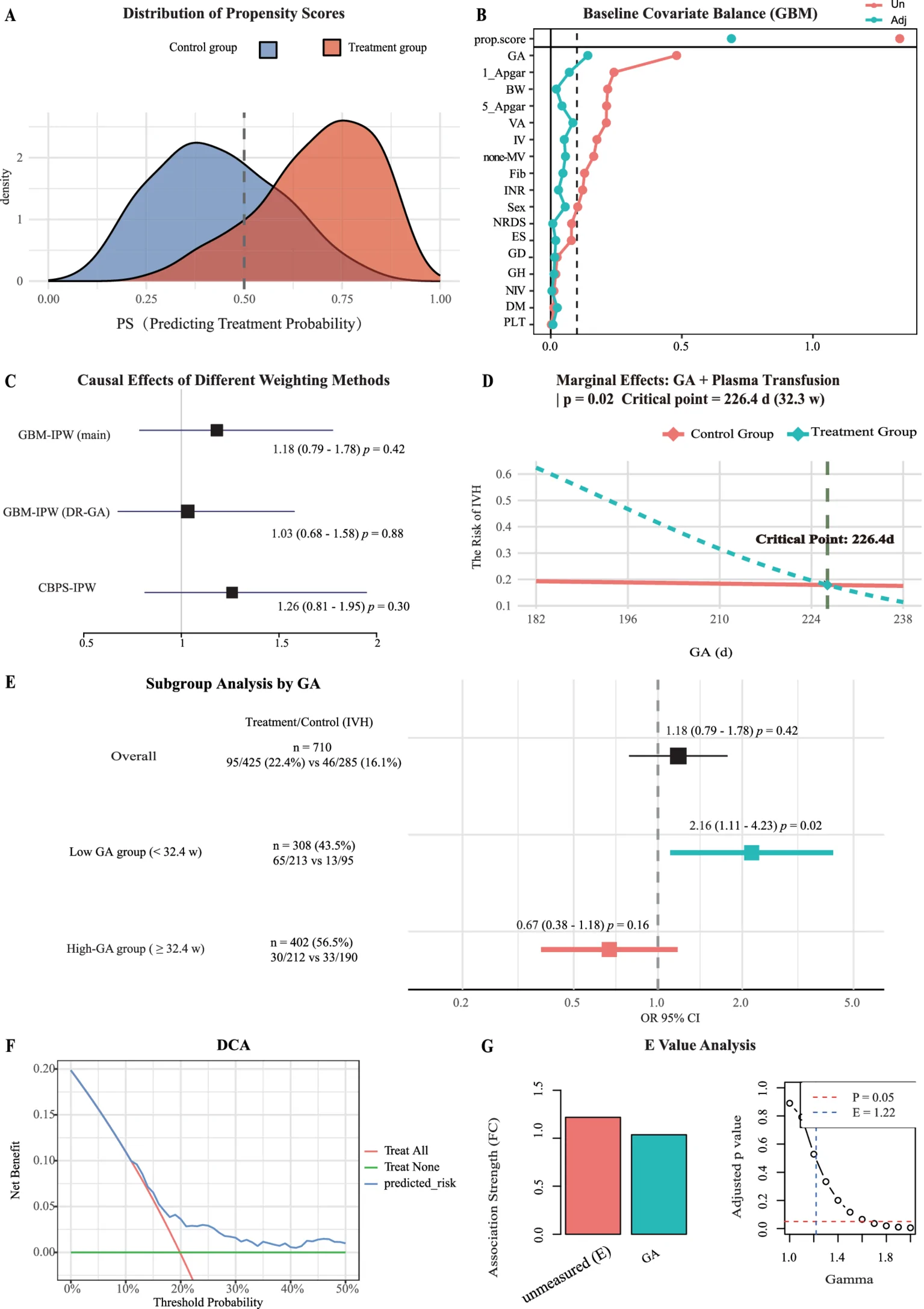
The inference and clinical utility evaluation of FFP transfusion on IVH in preterm infants. **(A)** Distribution of PS. Density plots of PS for the control (blue) and treatment (red) groups, with the vertical dashed line indicating the common support region. **(B)** Baseline covariate balance (GBM-IPW). SMD before (red, “Un”) and after (teal, “Adj”) GBM-IPW method. The vertical dashed line at 0.1 marks the threshold for acceptable balance. **(C)** Estimated effects of different weighting methods. OR and 95% CI for IVH associated with FFP transfusion, estimated using GBM-IPW (main analysis), GBM-DR restricted by adjusted GA, and CBPS-IPW. **(D)** Marginal effects of GA and FFP transfusion. Marginal risk of IVH in the control (red) and treatment (teal) groups, with the vertical dashed line highlighting the critical GA point (226.4 days/32.3 weeks). **(E)** Subgroup analyses of the association between FFP transfusion and IVH risk, stratified by GA at the optimal cutoff point of 32.4 weeks. ORs and 95% CIs were estimated using GBM-IPW. The overall effect (*n* = 710) and subgroup effects (low GA: *n* = 308; high GA: *n* = 402) are presented. **(F)** DCA for IVH risk prediction. Net benefit of model-guided FFP transfusion (blue) is compared to Treat-all (red) and Treat-none (green) strategies. The model outperforms Treat-all across clinically relevant thresholds. **(G)** The results of E value analysis. Left panel: comparison of the required unmeasured confounding (E-value) and the strength of known confounders, expressed as fold change in association. Right panel: sensitivity analysis showing the impact of increasing unmeasured confounding strength (*Gamma*) on the adjusted *p*-value. The red dashed line indicates the *p* = 0.05 significance threshold, and the blue dashed line marks the E-value of 1.22. BW, birth weight; CBPS-IPW, covariate balancing propensity score-inverse probability weight; CI, confidence intervals; DCA, decision curve analysis; DM, delivery mode; ES, early-onset sepsis; Fib, fibrinogen; FFP, fresh-frozen plasma; GA, gestational age; GBM-DR, gradient boosting machine-doubly robust; GBM-IPW, gradient boosting machine-inverse probability weight; GD, gestational diabetes; GH, gestational hypertension; INR, international normalized ratio; IV, invasive ventilation; IVH, intraventricular hemorrhage; NIV, non-invasive ventilation; non-MV, non-mechanical ventilation; NRDS, neonatal respiratory distress syndrome; OR, odds ratios; PLT, platelet; PS, propensity score; SMD, standardized mean differences; VA, vasoactive agents; VD, vaginal delivery.

Before weighting, the FFP and control groups differed significantly in GA, BW, sex, 1-minute Apgar score, 5-minute Apgar score, NRDS, ES, ventilation status (none, NIV, or IV), APTT, and vasoactive agent use (all *p* < 0.05). No significant differences were observed in delivery mode, GH, GDM, INR, Fib, or PLT (all *p*  > 0.05) (Table 1). The PS distributions showed substantial overlap within the common-support region, supporting the positivity assumption, although the separation between groups indicated confounding by indication (Figure 2A).

**Table 1:** Baseline characteristics of the population before and after propensity score and inverse probability weighting.

Characteristics	Overall	Control group	Treatment group	*p*
*n*	748	314	434	
GA, mean (SD)	225.87 (12.03)	229.07 (9.91)	223.55 (12.89)	<0.001
Weight, mean (SD)	1833.80 (556.14)	1901.33 (411.33)	1784.95 (636.86)	0.005
DM				0.798
VD	260 (34.8)	107 (34.1)	153 (35.3)	
CD	488 (65.2)	207 (65.9)	281 (64.7)	
Sex				0.007
Male	408 (54.5)	190 (60.5)	218 (50.2)	
Female	340 (45.5)	124 (39.5)	216 (49.8)	
Apgar-1, mean (SD)	8.49 (2.06)	8.77 (1.90)	8.29 (2.14)	0.001
Apgar-5, mean (SD)	9.25 (1.36)	9.41 (1.21)	9.13 (1.46)	0.005
GH (%)				0.457
No	673 (90.0)	279 (88.9)	394 (90.8)	
Yes	75 (10.0)	35 (11.1)	40 (9.2)	
GDM (%)				0.447
No	616 (82.4)	263 (83.8)	353 (81.3)	
Yes	132 (17.6)	51 (16.2)	81 (18.7)	
NRDS (%)				0.036
No	430 (57.5)	195 (62.1)	235 (54.1)	
Yes	318 (42.5)	119 (37.9)	199 (45.9)	
ES (%)				0.016
No	571 (76.3)	254 (80.9)	317 (73.0)	
Yes	177 (23.7)	60 (19.1)	117 (27.0)	
Ventilator (%)				<0.001
No ventilator use	179 (23.9)	105 (33.4)	74 (17.1)	
NIV	252 (33.7)	108 (34.4)	144 (33.2)	
IV	317 (42.4)	101 (32.2)	216 (49.8)	
APTT, mean (SD)	87.13 (15.20)	84.08 (13.74)	89.34 (15.83)	<0.001
INR, mean (SD)	1.45 (0.65)	1.40 (0.61)	1.48 (0.68)	0.104
Fib, mean (SD)	1.54 (0.67)	1.59 (0.68)	1.51 (0.66)	0.081
VA, mean (SD)	0.30 (0.46)	0.18 (0.38)	0.39 (0.49)	<0.001
PLT, mean (SD)	244.63 (66.16)	244.72 (69.68)	244.56 (63.57)	0.975
IVH (%)				0.067
No	602 (80.5)	263 (83.8)	339 (78.1)	
Yes	146 (19.5)	51 (16.2)	95 (21.9)	

APTT, activated partial thromboplastin time; CD, cesarean delivery; DM, delivery mode; ES, early-onset sepsis; Fib, fibrinogen; GA, gestational age; GDM, gestational diabetes mellitus; GH, gestational hypertension; INR, international normalized ratio; IV, invasive ventilation; NIV, non-invasive ventilation; NRDS, neonatal respiratory distress syndrome; VA, vasoactive agents; VD, vaginal delivery; IVH, intraventricular hemorrhage; PLT, platelet count.

GBM-IPW was applied to control baseline confounding bias. After GBM-IPW adjustment and trimming the extreme weights at the 2.5% quantile, a total of 38 outliers were excluded from the analysis, resulting in a sample size of 710 cases. Finally, 16 out of 17 covariates achieved balance (the absolute values of SMD < 0.1). The SMD of GA significantly decreased from 0.48 to 0.14 post-weighting, slightly above the 0.1 threshold (Figure 2B). GBM-DR estimation model with further adjustment for GA was performed to strengthen the robustness of results. The detailed baseline characteristics before and after weighting are shown in Figure 2B.

### 3.1 Overall estimated effect of FFP transfusion on IVH risk in preterm infants with prolonged APTT

A survey-weighted binomial generalized linear model via trimmed GBM-IPW weights was constructed to estimate the overall effect of FFP transfusion on IVH risk. The results showed FFP transfusion did not significantly reduce the IVH risk in the whole cohort (OR = 1.18; 95% CI: 0.79–1.78; *p* = 0.42). Except for GA, other covariates satisfied the criterion of absolute SMD < 0.1 To address this residual confounding, the GBM-DR model with additional adjustment for GA also revealed no significant association between FFP and IVH (adjusted OR = 1.03; 95% CI: 0.68–1.58; *p* = 0.88), consistent with the preliminary analysis.

We further applied CBPS method for sensitivity analysis. The main results remained consistent (adjusted OR = 1.26; 95% CI: 0.81–1.95; *p* = 0.30) with GBM-IPW method, indicating the robustness of our findings across weighting methods (Figure 2C).

To ameliorate the selection bias and assess the robustness, thirty-seven infants were excluded from the primary analytic cohort for observation ended within 72 h, including 2 deaths, 8 transfers, and 27 cases of withdrawal of intensive care at parental request. After reintroducing these infants, the inclusive landmark sensitivity cohort comprised 785 infants, with 449 in the FFP group and 336 in the control group. PS weighting ensured that all 17 baseline covariates were well balanced, with the largest absolute SMD of 0.027. Weight-adjusted logistic regression indicated no significant association between FFP transfusion and IVH, with an odds ratio of 1.172 (95% CI, 0.797–1.723; *p* = 0.420). Similarly, weighted Cox analysis produced a hazard ratio of 0.959 (95% CI, 0.679–1.355; *p* = 0.814) (Supplementary Figures 1, 2).

### 3.2 Effect modification of GA and subgroup analysis

Previous studies showed that GA considerably influence the occurrence of IVH (Egesa et al., 2021). Then, we aim to assess whether the effect of FFP transfusion on IVH is modified by GA. We added an interaction term of FFP exposure and centered GA to the IPW model. GA was mean-centered at 226.4 days to enhancethe interpretability of the interaction coefficient (Figure 2D). After adjusting for BW and sex, the interaction term (FFP × centered GA) was statistically significant (coefficient = −0.0436; 95% CI: −0.079 to −0.008; *p* = 0.02). These results suggest GA substantially modifies the relationship between FFP transfusion and IVH risk. The negative coefficient implies that FFP transfusion’s benefit attenuates and the harm increases with lower GA.

For clinical interpretability, the cohort was stratified at the mean GA (32.4w) into a low-GA group (<32.4w; *n* = 308) and a high-GA group (≥32.4w; *n* = 402). In the overall population (*n* = 710), FFP transfusion showed no statistically effect on IVH risk (OR = 1.18; 95% CI: 0.79–1.78; *p* = 0.42). However, in the low-GA group, FFP transfusion is harmful to IVH (OR = 2.16; 95% CI: 1.11–4.23; *p* = 0.02). In contrast, in the high-GA group, FFP transfusion tends to reduce the IVH’s occurrence (OR = 0.67; 95% CI: 0.38–1.18; *p* = 0.16), although this estimate did not reach conventional statistical significance (Figure 2E). Collectively, these findings identify the necessity of FFP transfusion based on GA stratification.

### 3.3 Clinical utility assessment via decision curve analysis

Researchers commonly performed DCA method to evaluate the clinical applicability of the predictive model and quantify net clinical benefit across a range ofthreshold probabilities (Suhas et al., 2023; VanderWeele and Ding, 2017). Universal FFP transfusion in the overall population generates negative net benefit across all clinically relevant thresholds. In contrast, within the threshold probability range of 10 to 35%, the model-guided selective intervention strategy consistently demonstrated greater net benefit than both the treat-all and treat-none strategy (Figure 2F), which improving net clinical benefit compared with non-selective regimens. This provides quantitative, evidence-based support for shifting from uniform FFP transfusion empirical practice toward individualized, risk-stratified strategy.

### 3.4 Sensitivity and robustness analysis

#### 3.4.1 E-value analysis

E-value analysis universally is used to examine the findings’ robustness and susceptibility (Blank et al., 2025; Haneuse et al., 2019). The E-value for association of FFP transfusion and IVH was 1.22. This value denotes the minimum strength of an unmeasured confounder-measured as risk ratios—required to fully explain the observed null effect (adjusted OR = 1.03). For context, the strongest measured confounder in the model GA had a corresponding risk ratio of approximately 1.18. Since the E-value (1.22) exceeds the observed effect of GA, the null finding is robust to unmeasured confounding with strength comparable to or weaker than GA (Figure 2G).

#### 3.4.2 Weight trimming sensitivity analysis

To identify the robustness of the findings, we attempt to evaluate the effect of extreme PS values between FFP and IVH through three sets of bilateral weight trimming (1, 2.5, and 5%, respectively). Different trimming methods showed the consistent results that FFP transfusion exerted no impact on IVH risk (OR range: 1.096–1.182; all 95% CI included 1.0; *p* > 0.05), further strengthening the robustness of main results yielded by TTE framework (Supplementary Table 2).

#### 3.4.3 Negative control analysis

Negative control analysis further identified confounding adjustment. The apparent association between FFP and lower BW observed in unadjusted analyses (–116.38 g; *p* = 0.005). While this difference reached statistical significance, it had limited clinical relevance and likely reflected residual confounding. Comparing BW distributions across the two cohorts, we found closely aligned curve peaks between 1,800 and 2,000 g, suggesting that extreme outliers largely drove this statistically significant finding. This result showed adequate control for GA–a domina (Supplementary Figure 3).

### 3.5 Analysis between FFP transfusion and IVH based on the causal forest model

**Figure 3:**
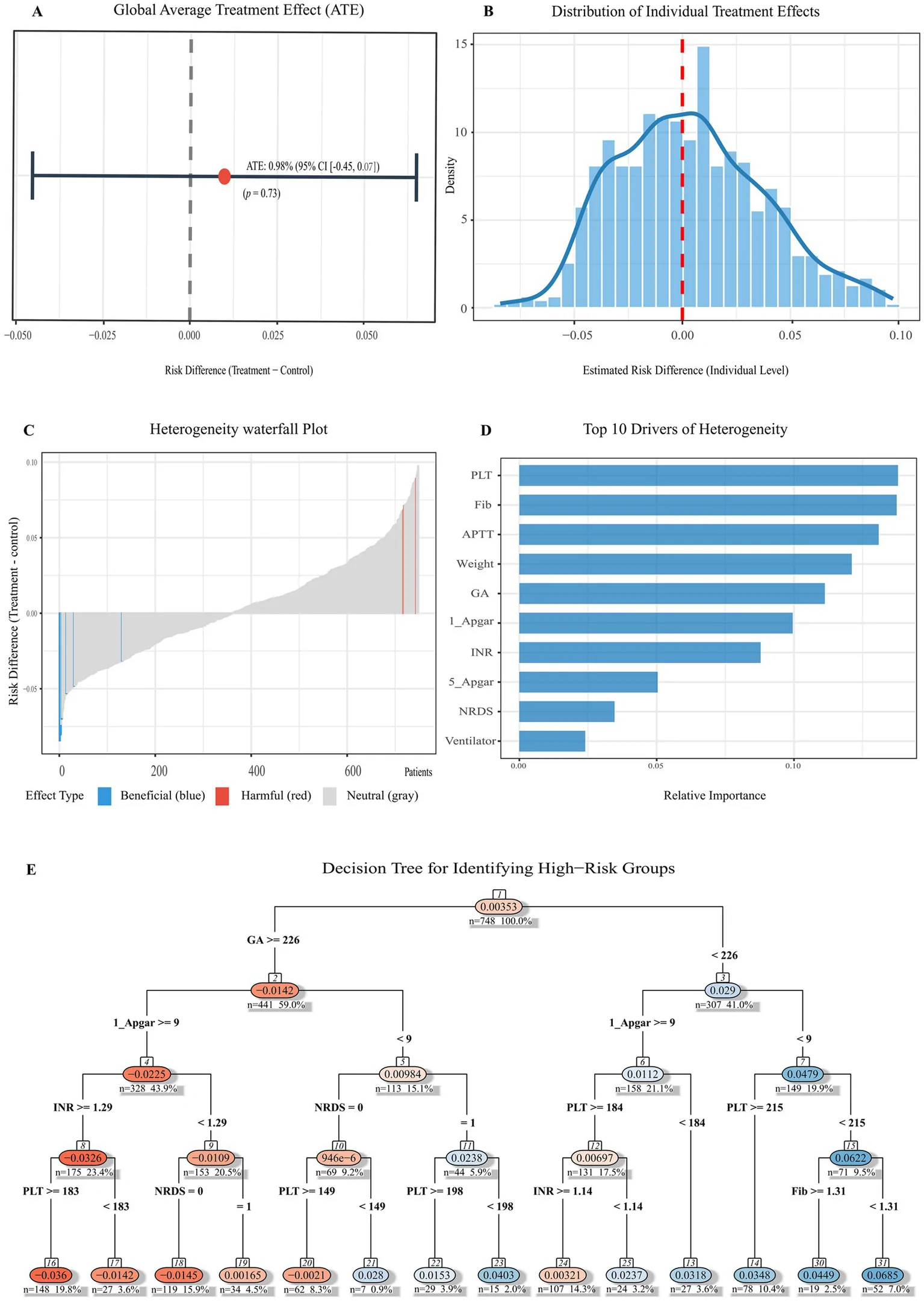
Integrated evaluation of FFP transfusion for IVH: causal effects, heterogeneity, and clinical decision support. **(A)** ATE of FFP transfusion on IVH risk. **(B)** Distribution of ITE for IVH risk. Density plot of estimated individual-level risk differences; most patients cluster near 0 (no treatment effect) with a slight distribution skew. **(C)** Heterogeneity waterfall plot of FFP transfusion effects on IVH risk. Patient-level risk differences sorted by estimated treatment effect; blue (beneficial), red (harmful), and grey (neutral) bars indicate effects with 95% CIs excluding or including 0, respectively. **(D)** Top 10 baseline characteristics driving treatment effect heterogeneity. Relative importance of key covariates (PLT, Fib, APTT, etc.) in modifying the FFP transfusion effect on IVH risk. **(E)** Decision tree for identifying IVH high-risk subgroups. Hierarchical subgroup stratification (by GA, Apgar-1, PLT, etc.) with corresponding risk differences and sample sizes (*n*, %) for each subgroup; values represent treatment effects of FFP transfusion on IVH risk. Due to the lack of an independent external validation cohort, the subgroups identified in panels **C, E** and the driver rankings in panel **D** are considered exploratory and hypothesis-generating. ATE, average treatment effect; APTT, activated partial thromboplastin time; FFP, fresh-frozen plasma; Fib, fibrinogen; GA, gestational age; ITE, individual treatment effects; INR, international normalized ratio; IVH, intraventricular hemorrhage.

Under the causal forest model, we determined the associations between FFP transfusion and IVH after adjusting the confounders, without post-treatment variables, across the overall population and individual patients. The overall ATE of FFP transfusion on IVH risk was 0.98%, with no significant difference (*p* = 0.73; 95% CI: −0.45–0.07) (Figure 3A), and we observed no clearly tendency of benefit or adverse effects through the ITE distribution (Figure 3B). According the waterfall plot, only a small subset of participants showed treatment response, highlighting substantial interindividual variability (Figure 3C).

Through the covariate feature importance analysis, we uncovered the key baseline roles associated with varying treatment effects: PLT, Fib, APTT, BW, GA, 1-min Apgar score, INR, 5-min Apgar score, NRDS, and ventilator (Figure 3D). The decision tree approach further stratified high-risk group of FFP transfusion based on variables such as GA, 1-min Apgarscore, INR, PLT, and NRDS. Certain subgroups appeared to benefit from FFP transfusion, whereas the major part experienced minimal effects or even adverse outcomes (Figure 3E). In conclusion, these results does not support routine FFP transfusion for the prevention of neonatal IVH. For the absence of external validation, these results are interpreted as exploratory and hypothesis-generating founding and more studies are needed.

## 4 Discussion

Using retrospective real-world clinical data, we constructed an integrated analytical approach combining TTE and machine learning. We formalized all core components of the TTE framework, then deployed gradient GBM-IPW to balance baseline clinical covariates and replicate the analytical structure of a randomized controlled trial. This finding comprehensively determined the estimated effect of FFP transfusion on IVH risk among 748 preterm neonates with prolonged APTT, employing multiple causal inference methods, individualized effect estimation and sensitivity analyses. Consistent results generated from multiple weighting approaches and ATE evaluation showed no significant overall association between FFP transfusion and IVH at the population level. Further analyses confirmed GA as a prominent effect modifier. FFP transfusion correlated with elevated IVH risk in extremely immature preterm infants, while no significant beneficial or harmful effects were observed in infants closer to 34 weeks’ gestation. Weight trimming, E-value analysis and negative control testing all validated the robustness of our primary outcomes. The 24-h landmark sensitivity analysis produced results consistent with the primary analysis and eased concerns that immortal time bias influenced our findings. This analysis nonetheless only evaluated associations among infants alive, hospitalized, and IVH-free at 24 h after baseline, and could not detect IVH events arising within the first 24 h. Using causal forest modelling, we further identified marked variability in how individual infants responded to FFP transfusion, with PLT, Fib, APTT and BW emerging as the key factors underlying these divergent treatment effects. DCA additionally identified model-guided selective intervention yielded superior net clinical benefit compared with universal FFP transfusion. These results support the adoption of individualized, risk-stratified transfusion strategies in neonatal clinical practice.

To date, there remains a controversy regarding whether the FFP transfusion can reduce the risk of IVH by correcting coagulation abnormalities. Our results clearly indicated that FFP transfusion yielded no overall clinical benefit, alongside striking heterogeneity in efficacy based on GA. A multicenter observational study conducted by Kuperman et al. also demonstrated that empirical FFP transfusion failed to lower bleeding risk among preterm infants with coagulation abnormalities and could raise the occurrence of adverse events (Iacobucci et al., 2016). Authoritative guidelines from the American Association of Blood Banks (AABB) and the Egyptian Neonatal Transfusion Guidelines (Christensen et al., 2014a; Vickers et al., 2016), strongly discouraged routine FFP transfusion in preterm infants with isolated coagulation abnormalities in the absence of active bleeding. These recommendations were fully consistent with the evidence generated by our study. Discrepancies across prior published studies stem primarily from unmeasured confounding by indication. Critically, infants with more severe clinical disease were more likely to receive FFP transfusion while also carrying an inherently higher risk of IVH, which generated misleading correlational signals (Neary et al., 2013; Harrell, 2015). Our study conducted a strict TTE framework to effectively mitigate this critical bias and enhance the validity and robustness of our findings.

We found no overall clinical advantages of prophylactic FFP, a result consistent with the established physiological immaturity of neonatal coagulation. Newborns inherently possess an underdeveloped coagulation cascade. Prolonged APTT in preterm infants primarily stemmed from physiological deficiencies of coagulation factors II, VII, and IX, rather than pathological coagulation disorders (Li et al., 2022). FFP transfusion was primarily indicated for bleeding secondary to pathological coagulation factor deficiencies, and it showed limited efficacy in correcting the physiological immaturity of the neonatal coagulation system (Lu et al., 2025). Beyond its limited value for correcting physiological coagulation immaturity, FFP contains coagulation factors at normal adult plasma concentrations, and its therapeutic effect was fundamentally constrained by the neonatal blood volume and dilution effects following transfusion (Tyagi et al., 2022). The rise in circulating coagulation factor levels after transfusion remains modest and fails to produce any clinically meaningful reduction in IVH risk. Our results support the clinical principle that FFP transfusion should not be given merely for isolated abnormal coagulation parameters in the absence of active bleeding. These findings held significant implications for curbing the inappropriate use of FFP in neonatal clinical practice.

A core novelty of our study is the identification of striking GA-related variation in FFP’s ability to prevent IVH in preterm infants. In short, lower GA corresponds to elevated risks of adverse events after FFP administration. In contrast, FFP exhibited neutral or modest protective effects only in infants close to 34 weeks’ gestation. Subgroup analyses, marginal effect modeling and interaction tests fully validated this conclusion. Our findings provided robust evidence for individualized transfusion decision-making in clinical practice. Stratified analyses by GA demonstrated a progressive shift in how FFP transfusion correlates with IVH risk. Extremely preterm infants below 28 weeks showed a marked increase in IVH risk. Fresh‑frozen plasma transfusion might increase the risk of IVH in the lower‑gestational‑age subgroup, whereas a trend toward reduced IVH risk is observed in the higher‑gestational‑age subgroup. Previous study have suggested that prophylactic transfusion of FFP could reduce the risk of IVH in extremely preterm infants. Those studies exclusively enrolled infants with a GA of 23 to 26 weeks and failed to conduct subgroup analyses stratified by GA (Dani et al., 2009). Our study enrolled patients with a broader range of GA and yielded more generalizable findings. Furthermore, this is also the first study worldwide to specifically address this underexplored clinical issue. Marginal effect plots further illustrated that at approximately 180 days of GA (<28 weeks), the predicted IVH risk in the FFP transfusion group was significantly higher than that in the control group. The two curves crossed at approximately 226.4 days (≈ 32.3 weeks). Above this gestational cutoff, model-predicted IVH risk among infants receiving FFP falls below that of the control cohort (Adeshina et al., 2026; Wu et al., 2020; Kuperman et al., 2015).

This heterogeneity correlated closely with the developmental maturation of the preterm infant’s brain and coagulation system (Kuperman et al., 2015). Infants with lower GA had more fragile germinal matrix vasculature and their cerebral endothelial cells were more susceptible to injury (Christensen et al., 2014a; Sokou et al., 2022). Their physiological coagulation deficiencies were more marked resulting in a higher baseline risk of IVH. FFP transfusion could rarely fully correct coagulation dysfunction; instead, excessive hemolysis during the infusion process and secondary microthrombosis may aggravate the existing bleeding tendency or produce fluctuations in cerebral blood flow and venous pressure, further increasing the risk of bleeding (Adeshina et al., 2026; Lee et al., 2011; Wei et al., 2025).

As GA increases, the cerebral vessels and coagulation system of premature infants gradually mature, lowering the baseline risk of intracranial hemorrhage. The potential benefits of FFP transfusion might increase and a trend toward potential protective effects might emerge in more mature preterm infants. These results suggest that GA serves as a key stratification indicator, and clinicians need to comprehensively consider the other baseline characteristics when determining the indication for FFP infusion. Empirical FFP transfusion should be strictly avoided in extremely preterm infants and decisions for more mature preterm infants required rigorous risk–benefit evaluation (Tyagi et al., 2022). This conclusion aligned with findings from prior investigations.

We performed feature importance analysis and identified the top 10 baseline predictors associated with heterogeneity in the therapeutic effect of FFP transfusion. Ranked by relative importance, these indicators were: PLT, Fib, APTT, BW, GA, 1-min Apgar score, INR, 5-min Apgar score, NRDS, and MV. These variables correspond to well-established risk factors for IVH among preterm infants and core markers reflecting global neonatal illness severity (Houben et al., 2020; Wei et al., 2025). These variables exert modifying effects on FFP-related outcomes, illustrating how baseline clinical status shapes treatment response. For example, PLT, a key determinant of hemostasis, facilitated platelet aggregation and functions synergistically with circulating coagulation factors. Infants with lower PLT levels exhibit more severe hemostatic impairment and might therefore derive greater benefit from FFP transfusion (Christensen et al., 2014a). In contrast, indicators such as NRDS and MV reflected respiratory status and illness severity. As IVH arises from multiple interacting factors, correcting isolated coagulation defects alone cannot lower hemorrhage risk, which restricts the clinical benefits of FFP transfusion. Collectively, these results suggest clinicians should not base FFP transfusion decisions merely on abnormal coagulation indices like prolonged APTT. Instead, a comprehensive clinical decision-making process for FFP transfusion necessitates a multi-dimensional assessment integrating multiple baseline indicators, rather than formulating a plan solely based on a single indicator (Houben et al., 2025). Notably, all these core indicators are routinely measured in clinical practice and are easily obtainable. This provided a solid foundation for the future development of a clinically applicable, individualized transfusion risk stratification model with substantial translational potential in real-world neonatal care.

The main methodological strength of this study lay in its rigorous TTE framework integrated with causal forest analysis. This novel method enhanced the reliability of estimating the effect of FFP infusion on IVH, and also provided a highly valuable methodological paradigm for subsequent clinical studies. First, the TTE framework was adopted to replicate the design of RCTs using observational data (McCormick et al., 2025). This approach effectively mitigated the main methodological issue of confounding bias and separated the therapeutic effect of FFP from the inherent baseline status of patients. Accordingly, the study findings were more consistent with the true relationship between the intervention and the outcome. Second, before fitting the GBM to estimate PS and to perform IPW using 17 key confounders, we evaluated the PS distribution between the two groups and confirmed only partial overlap. The subsequent baseline balance analysis revealed that covariates exhibited both upward and downward shifts in SMD, which points to prominent clinical heterogeneity within this cohort of preterm neonates admitted to the NICU. This complexity is the key reason for the scarcity of related studies – most existing studies are limited to correlation analyses and struggle to address the issue of confounding factors. Through the aforementioned statistical approaches, this study minimized bias during analysis and strengthened the robustness of the findings (Lee et al., 2021; Choi et al., 2020; Haneuse et al., 2019).

We performed sensitivity analyses (including weight trimming, E-value analysis and negative BW controls) to evaluate the influence of unmeasured confounders. Although these analyses suggested that there might be unmeasured residual confounding bias in the study, multiple robustness tests (including alternative weighting specifications and stratified subgroup analyses) consistently confirmed the robustness of the primary conclusions. For instance, we conducted a negative control analysis using BW, a baseline metric measured before intervention and unaffected by FFP transfusion. Without adjustment for GA, the mean BW in the FFP group was 116.38 g lower than that in the control group. Although this difference was statistically significant, it had limited clinical relevance and might have reflected residual confounding. When contrasting BW distributions between the two cohorts, we observed closely matched curve peaks ranging from 1,800-2,000 g, which implied that extreme outliers largely accounted for the statistically significant result.

After adjustment for GA, the GBM-DR model indicated that the association lost statistical significance, demonstrating that the observed differences in BW were primarily attributable to variation in GA rather than the treatment itself. This observation validated our GA adjustment strategy and reinforced the main conclusion that FFP transfusion offers no protection against IVH in preterm infants. Meanwhile, machine learning approaches overcame the limitations of conventional analytical models, which only estimated population-averaged treatment effects. These techniques enabled a shift from population-level inference to individualized, precision-targeted interventions (Mangold et al., 2021). Feature importance analysis identified 10 core baseline indicators. Decision-tree modeling yielded clinically applicable stratification strategies, and heterogeneous waterfall plots illustrated the varying individual effects of FFP transfusion. These complementary analytic outputs delivered intuitive, actionable evidence to support personalized clinical decision-making (Saeedi et al., 2025; Mangold et al., 2021; Xie et al., 2025). The integration of TTE and machine learning mitigated observational data biases, improving the accuracy of individualized neonatal research.

We aim to provide robust evidence about the clinical guidance of FFP transfusion in preterm neonates with coagulopathy and identify the subgroup who truly benefit from FFP. Currently, most transfusion guidelines for preterm infants rely only on BW or GA to define transfusion indications and exclude risk stratification for complications (Christensen et al., 2014a; Haghshenas-Mojaveri et al., 2024; Scrivens et al., 2023). Based on our decision tree and DCA model, individualized FFP transfusion strategies should integrate multiple clinical factors, such as GA, Apgar scores, rather than a uniform guideline. Specifically, routine FFP transfusion is not only inadvisable for infants with isolated prolonged APTT without active bleeding, but may also cause harm to the extremely preterm infants (<28 weeks’ GA). It’s essential to comprehensively assess baseline indicators including GA, PLT, Fib, Apgar score, and underlying clinical conditions together for the precise strategies. Chen’s team identified PLT and PLT transfusion were closely related with the incidence of IVH and significantly increased death rate (Chen et al., 2022). However, regarding Apgar scores, one study was similar to our results that Apgar scores was a predictive factor of IVH, whereas another study showed they have no association with long-term neurological complications (Ehrhardt et al., 2026; Ehrhardt et al., 2023). Furthermore, in order to improve the clinical outcomes of this vulnerable population, clinicians should combine FFP transfusion with standardized routine nursing care and symptomatic supportive treatment to reduce the initial risks of IVH.

Nevertheless, this study presents several inherent limitations. First, we strictly enrolled only preterm infants with an initial APTT ≥70 s upon coagulation testing, whereas infants presenting with active hemorrhage (including gastrointestinal and pulmonary hemorrhage) at study entry were excluded. Approximately 1,000 infants who developed initial IVH or caused by other factors during the study period were excluded from analyses. Given that the primary research question focused on the preventive effect of FFP transfusion on IVH, these eligibility criteria were reasonable constraints inherent to the study design. These limitations do not undermine the specificity or reliability of the conclusions, but merely limit the generalizability of the findings to cohorts receiving non-prophylactic FFP transfusion. On the other hand, larger samples are needed in the future to identify this result across broader APTT thresholds like greater than 60 s or 80s.

Based on the results presented here, future research may address several key research priorities outlined below. Primarily, multicenter prospective cohort studies are needed to externally validate the generalizability and clinical utility of the decision-tree-based stratification model. Such follow-up work can incorporate additional unmeasured confounders to refine model performance and improve its practical value in routine clinical practice. Second, RCTs could recruit more participants from potentially beneficial subgroups (GA > 32.4 weeks) as well as critically ill neonates with active bleeding to directly evaluate the efficacy of FFP transfusion. Third, it’s beneficial to explore the optimal dosage, infusion timing, and administration strategies for FFP according to baseline characteristics. Fourth, purified coagulation factor concentrates and other hemostatic interventions warrant further investigation as substitutes for standard FFP, given their potential to lower transfusion-related complications and align better with neonatal physiology characteristics. Fifth, defining GA-appropriate reference intervals for neonatal coagulation markers is essential to distinguish normal developmental immaturity from true coagulopathy, which could reduce unnecessary FFP transfusions in clinical practice.

## 5 Conclusion

According a rigorous TTE framework, this study revealed that FFP transfusion failed to lower overall IVH risk in preterm infants with prolonged APTT by diverse causal inference approaches and sensitivity analyses. GA was further identified a estimated protective factor of IVH and markedly modified the effect of FFP transfusion. Premature infants with lower GA accepted FFP transfusion significantly has a higher IVH risk, while those more mature neonates showed a mild trend toward reduced risk. High-risk subgroups about FFP transfusion can identified by GA, Apgar scores, and coagulopathy factors. In general, risk stratification and personalized FFP transfusion strategies are recommended for clinical use when meeting prolonged APTT rather than routine transfusion. Multicenter trials are required to confirm our findings, polish GA-based transfusion standards and avoid transfusion complications.

## Data Availability

The datasets presented in this study can be found in online repositories. The names of the repository/repositories and accession number(s) can be found in the article/Supplementary material.
