# Correction: Effect of fresh-frozen plasma transfusion on intraventricular hemorrhage risk in preterm infants with prolonged APTT: a target trial emulation with causal forest analysis

**DOI:** 10.3389/fnins.2026.1995110

**Published:** 2026-09-24

**Authors:** Bingyi Tang, Qinyi Yu, Jialin Zhang, Xinyu Duan, Teng Meng, Zhengdong Chen, Qingqi Ran, Yi Zhang, Siqi Tian, Zhangxue Hu, Wenjie Peng

**Affiliations:** 1 Department of Pediatrics, Daping Hospital, Army Medical University, Chongqing, China; 2 Department of Breast and Thyroid Surgery, Daping Hospital, Army Medical University, Chongqing, China; 3 Operation Center of Huashan Hospital, Fudan University, Shanghai, China

**Keywords:** fresh-frozen plasma, intraventricular hemorrhage, preterm infants, prolonged activated partial thromboplastin time, target trial emulation

An incorrect number was provided for the National Natural Science Foundation of China Young Scientist Fund. The correct number is 82201908.

The original version of this article has been updated.

