# Supplementary material for: Effect of fresh-frozen plasma transfusion on intraventricular hemorrhage risk in preterm infants with prolonged APTT: a target trial emulation with causal forest analysis
Source: Front Neurosci. 2026 Sep 7;20:1950034. doi: 10.3389/fnins.2026.1950034 (PMC13590724; doi:10.3389/fnins.2026.1950034)
Supplement: Supplementary file 1 [file Data_Sheet_1.PDF]

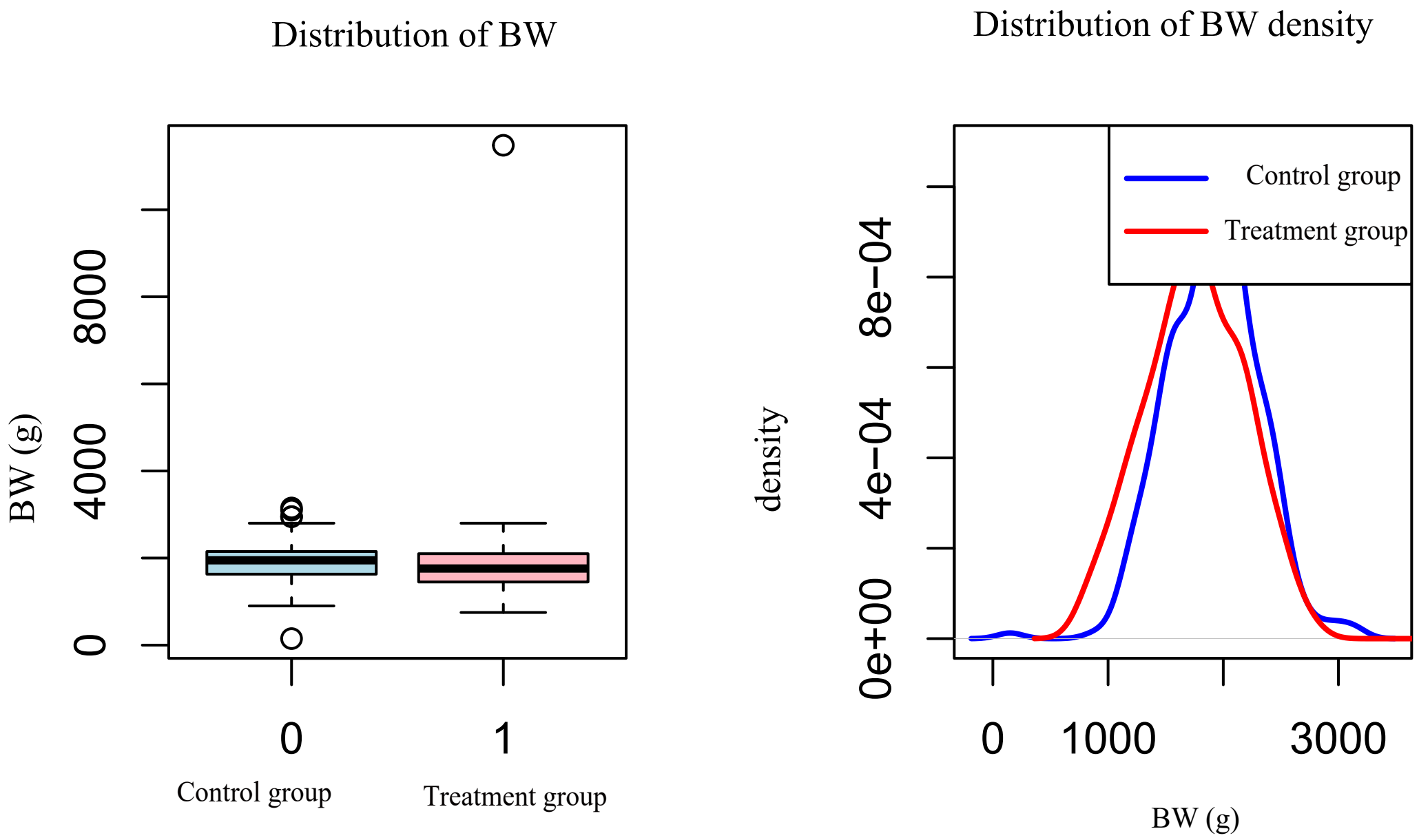

Distribution of BW
BW (g)
0
4000
8000
0
1
Control group
Treatment group
Distribution of BW density
density
0e+00
4e−04
8e−04
Control group
Treatment group
0
1000
3000
BW (g)
