# Supplementary material for: Effect of fresh-frozen plasma transfusion on intraventricular hemorrhage risk in preterm infants with prolonged APTT: a target trial emulation with causal forest analysis
Source: Front Neurosci. 2026 Sep 7;20:1950034. doi: 10.3389/fnins.2026.1950034 (PMC13590724; doi:10.3389/fnins.2026.1950034)
Supplement: Supplementary file 2 [file Data_Sheet_2.PDF]

Covariate Balance in the 24 hour Landmark Analysis

Logistic propensity score weighting for the average treatment effect (ATE)

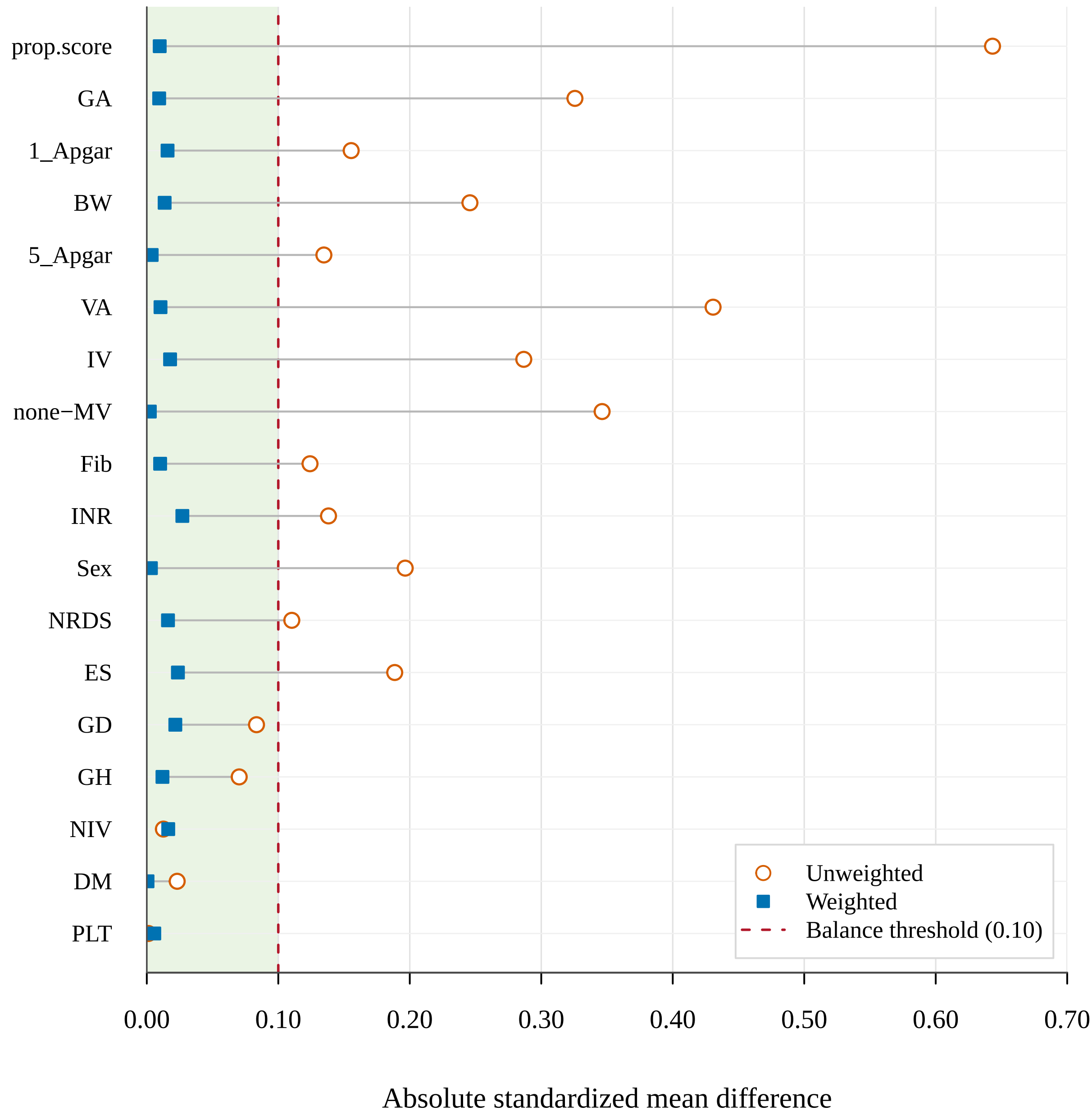

All 17 covariates achieved |SMD| < 0.10 after weighting; maximum weighted |SMD| = 0.027. n = 785.
