# Supplementary material for: Effect of fresh-frozen plasma transfusion on intraventricular hemorrhage risk in preterm infants with prolonged APTT: a target trial emulation with causal forest analysis
Source: Front Neurosci. 2026 Sep 7;20:1950034. doi: 10.3389/fnins.2026.1950034 (PMC13590724; doi:10.3389/fnins.2026.1950034)
Supplement: Supplementary file 3 [file Data_Sheet_3.PDF]

Effect Estimates in the 24−hour Landmark Sensitivity Analysis

Logistic propensity score weighting for the average treatment effect (ATE)

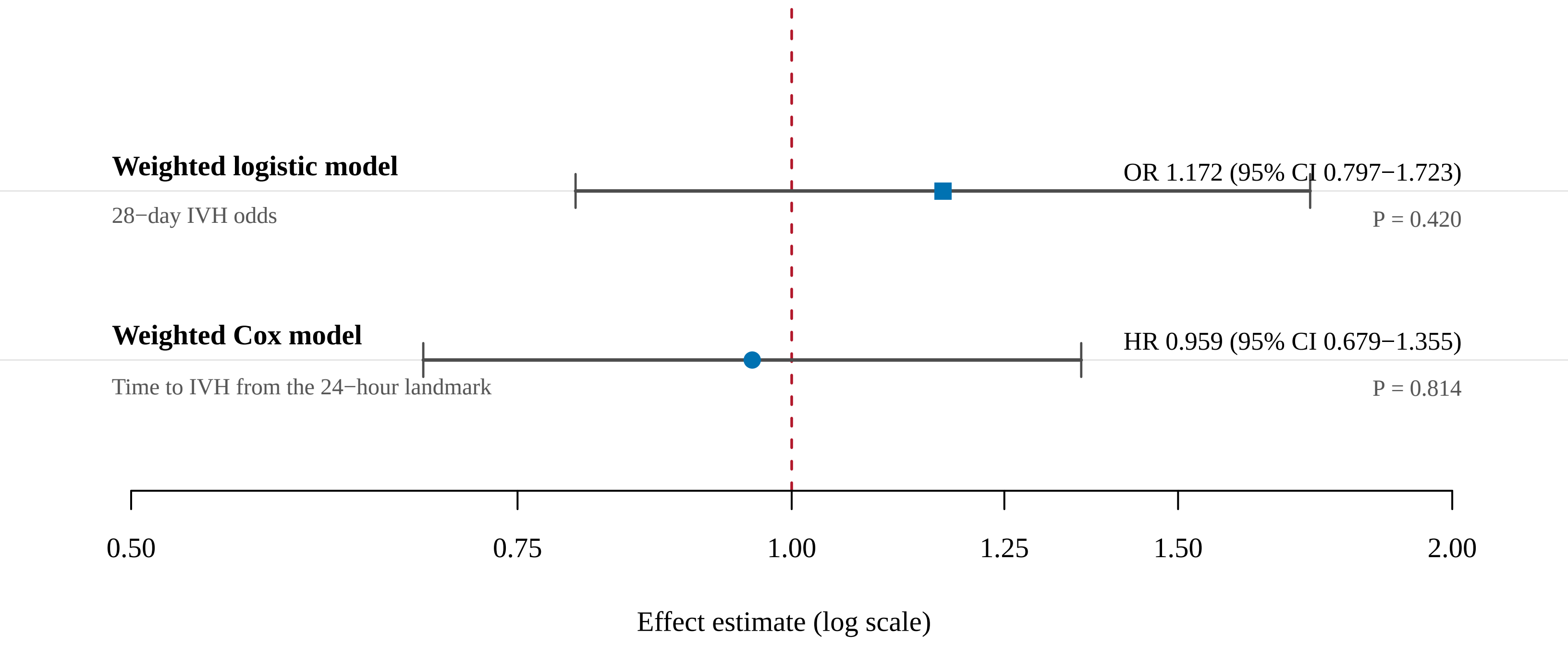

The vertical dashed line indicates the null value (OR or HR = 1.00). Landmark cohort: n = 785.
