# Supplementary material for: Effect of fresh-frozen plasma transfusion on intraventricular hemorrhage risk in preterm infants with prolonged APTT: a target trial emulation with causal forest analysis
Source: Front Neurosci. 2026 Sep 7;20:1950034. doi: 10.3389/fnins.2026.1950034 (PMC13590724; doi:10.3389/fnins.2026.1950034)
Supplement: Supplementary file 4 [file Data_Sheet_5.DOCX]

Codes and R packages required for the experiment:

1. The procedure of Target Trial Emulation.

if (!require("WeightIt")) install.packages("WeightIt")

if (!require("cobalt")) install.packages("cobalt")

if (!require("dplyr")) install.packages("dplyr")

if (!require("survey")) install.packages("survey")

library(WeightIt)

library(cobalt)

library(dplyr)

library(survey)

covariates <- c("GW", "Weight", "DM", "Sex", "X1_Apgar", "X5_Apgar",

"GH", "GD", "NRDS", "ES", "Ventilator", "INR", "Fib", "VA", "PLT")

treatment_var <- "Plasma"

outcome_var <- "IVH"

cat("\n=== Variable type conversion ===\n")

my_dataframe$DM <- factor(my_dataframe$DM)

my_dataframe$Sex <- factor(my_dataframe$Sex)

my_dataframe$GH <- factor(my_dataframe$GH)

my_dataframe$GD <- factor(my_dataframe$GD)

my_dataframe$NRDS <- factor(my_dataframe$NRDS)

my_dataframe$ES <- factor(my_dataframe$ES)

my_dataframe$VA <- factor(my_dataframe$VA)

my_dataframe$Plasma <- factor(my_dataframe$Plasma)

my_dataframe$IVH <- factor(my_dataframe$IVH)

my_dataframe$Ventilator <- factor(my_dataframe$Ventilator,

levels = c(0, 1, 2),

ordered = TRUE)

correct_covariates <- c("GW", "Weight", "DM", "Sex", "X1_Apgar", "X5_Apgar",

"GH", "GD", "NRDS", "ES", "Ventilator", "INR", "Fib", "VA", "PLT")

cat("\n=== Variable type checking ===\n")

var_types <- sapply(my_dataframe[, correct_covariates], class)

print(var_types)

formula_gbm <- as.formula(paste(treatment_var, paste(covariates, collapse = " + "), sep = " ~ "))

cat("formula：\n")

print(formula_gbm)

cat("\n=== GBM weighting ===\n")

set.seed(123)

weight_gbm <- weightit(

formula = formula_gbm,

data = my_dataframe,

method = "gbm",

estimand = "ATE",

stabilize = TRUE,

n.trees = 2000,

interaction.depth = 2,

criterion = "smd.mean"

)

my_dataframe$weights_gbm <- weight_gbm$weights

cat("GBM weighting ending！\n")

cat("weighting range：", round(range(weight_gbm$weights), 4), "\n")

cat("weighting mean value：", round(mean(weight_gbm$weights), 4), "\n")

cat("\n=== weighting trimming ===\n")

lower_trim <- quantile(weight_gbm$weights, 0.025, na.rm = TRUE)

upper_trim <- quantile(weight_gbm$weights, 0.975, na.rm = TRUE)

cat("Lower trim limit (2.5th percentile)：", round(lower_trim, 4), "\n")

cat("Upper trim limit (97.5th percentile)：", round(upper_trim, 4), "\n")

my_dataframe$weights_trimmed <- ifelse(

weight_gbm$weights < lower_trim | weight_gbm$weights > upper_trim,

NA,

weight_gbm$weights

)

my_dataframe_trimmed <- my_dataframe[!is.na(my_dataframe$weights_trimmed), ]

cat("Original sample size：", nrow(my_dataframe), "\n")

cat("Sample size after trimming：", nrow(my_dataframe_trimmed), "\n")

cat("Proportion of trimmed samples：", round((nrow(my_dataframe) - nrow(my_dataframe_trimmed)) / nrow(my_dataframe) * 100, 2), "%\n")

cat("Weight range after trimming：", round(range(my_dataframe_trimmed$weights_trimmed, na.rm = TRUE), 4), "\n")

cat("\n=== Covariate balance assessment ===\n")

balance_result <- bal.tab(

weight_gbm,

stats = c("mean.diffs"),

thresholds = c(m = 0.1),

un = TRUE,

disp = c("means", "sds")

)

print(balance_result)

cat("\n=== Covariate balance visualization ===\n")

\# Love plot

love.plot(weight_gbm,

threshold = 0.1,

abs = TRUE,

var.order = "unadjusted",

line = TRUE,

title = "GBMBalance after propensity score weighting",

subtitle = paste("sample size：", nrow(my_dataframe_trimmed)))

cat("\n=== Estimation of weighted treatment effects ===\n")

design_trimmed <- svydesign(

ids = ~1,

weights = ~weights_trimmed,

data = my_dataframe_trimmed

)

model_weighted <- svyglm(

as.formula(paste(outcome_var, "~", treatment_var)),

design = design_trimmed,

family = binomial()

)

summary_model <- summary(model_weighted)

cat("\n=== Results of weighted logistic regression ===\n")

print(summary_model)

or_value <- exp(coef(model_weighted)[paste0(treatment_var, "1")])

ci_value <- exp(confint(model_weighted)[paste0(treatment_var, "1"), ])

p_value <- summary_model$coefficients[paste0(treatment_var, "1"), "Pr(>|t|)"]

cat("\n=== main treatment effect ===\n")

cat("ORvalue：", round(or_value, 3), "\n")

cat("95% CI：[", round(ci_value[1], 3), ", ", round(ci_value[2], 3), "]\n")

> summary_model <- summary(model_weighted)

> coef_mat <- summary_model$coefficients

> p_value <- coef_mat[paste0(treatment_var, "1"), "Pr(>|t|)"]

> cat("P value：", format.pval(p_value, digits = 3), "\n")

cat("=== Basic distribution of GW ===\n")

cat("sample size：", length(my_dataframe$GW), "\n")

cat("missing value：", sum(is.na(my_dataframe$GW)), "\n")

cat("mean ± SD：", round(mean(my_dataframe$GW, na.rm = TRUE), 2),

"±", round(sd(my_dataframe$GW, na.rm = TRUE), 2), "\n")

cat("IQR：", round(median(my_dataframe$GW, na.rm = TRUE), 2),

"（", round(quantile(my_dataframe$GW, 0.25, na.rm = TRUE), 2),

"-", round(quantile(my_dataframe$GW, 0.75, na.rm = TRUE), 2), "）\n")

cat("range：", round(min(my_dataframe$GW, na.rm = TRUE), 2),

"-", round(max(my_dataframe$GW, na.rm = TRUE), 2), "\n")

cat("\n=== GW distribution by treatment group ===\n")

library(dplyr)

gw_summary <- my_dataframe %>%

group_by(Plasma) %>%

summarise(

n = n(),

mean = round(mean(GW, na.rm = TRUE), 2),

sd = round(sd(GW, na.rm = TRUE), 2),

median = round(median(GW, na.rm = TRUE), 2),

Q1 = round(quantile(GW, 0.25, na.rm = TRUE), 2),

Q3 = round(quantile(GW, 0.75, na.rm = TRUE), 2),

min = round(min(GW, na.rm = TRUE), 2),

max = round(max(GW, na.rm = TRUE), 2)

) %>%

mutate(Group = ifelse(Plasma == 0, "control group", "treatment group"))

print(gw_summary)

cat("\n=== Between-group difference test ===\n")

shapiro_test <- shapiro.test(my_dataframe$GW)

cat("Shapiro-WilkNormality test：W =", round(shapiro_test$statistic, 4),

"，p =", format.pval(shapiro_test$p.value, digits = 3), "\n")

if (shapiro_test$p.value > 0.05) {

t_test <- t.test(GW ~ Plasma, data = my_dataframe, var.equal = FALSE)

cat("two-sample ttest：t =", round(t_test$statistic, 3),

"，p =", format.pval(t_test$p.value, digits = 3), "\n")

} else {

wilcox_test <- wilcox.test(GW ~ Plasma, data = my_dataframe)

cat("Mann-Whitney Utest：W =", round(wilcox_test$statistic, 3),

"，p =", format.pval(wilcox_test$p.value, digits = 3), "\n")

}

> par(mfrow = c(1, 2))

> boxplot(GW ~ Plasma, data = my_dataframe,

+	main = "GW distribution（days）",

+	xlab = "treatment group",

+	ylab = "GA（day）",

+	col = c("lightblue", "lightgreen"),

+	names = c("control group", "treatment group"))

> my_dataframe$GW_weeks <- my_dataframe$GW / 7

> boxplot(GW_weeks ~ Plasma, data = my_dataframe,

+	main = "GWdistribution（weeks）",

+	xlab = "treatment group",

+	ylab = "GA（week）",

+	col = c("lightblue", "lightgreen"),

+	names = c("control group", "treatment group"))

>

> par(mfrow = c(1, 1))

library(survey)

design_2.5 <- svydesign(

ids = ~1,

weights = ~weights_trimmed,

data = my_dataframe_trimmed

)

model_main <- svyglm(

IVH ~ Plasma,

design = design_2.5,

family = binomial()

)

model_dr <- svyglm(

IVH ~ Plasma + GW,

design = design_2.5,

family = binomial()

)

cat("=== main analysis results ===\n")

summary(model_main)

cat("\n=== Doubly robust estimation（adjusting GW）===\n")

summary(model_dr)

or_main <- exp(coef(model_main)["Plasma1"])

ci_main <- exp(confint(model_main)["Plasma1", ])

or_dr <- exp(coef(model_dr)["Plasma1"])

ci_dr <- exp(confint(model_dr)["Plasma1", ])

cat("main agalysis（unadjusted GW）：\n")

cat("OR = ", round(or_main, 3), " (95% CI: ", round(ci_main[1], 3), "-", round(ci_main[2], 3), ")\n")

cat("P = ", round(summary(model_main)$coefficients["Plasma1", "Pr(>|t|)"], 4), "\n\n")

cat("dr（adjusted GW）：\n")

cat("OR = ", round(or_dr, 3), " (95% CI: ", round(ci_dr[1], 3), "-", round(ci_dr[2], 3), ")\n")

cat("P = ", round(summary(model_dr)$coefficients["Plasma1", "Pr(>|t|)"], 4), "\n\n")

or_gw <- exp(coef(model_dr)["GW"])

ci_gw <- exp(confint(model_dr)["GW", ])

cat("GW’ effect（increased per day）：\n")

cat("OR = ", round(or_gw, 3), " (95% CI: ", round(ci_gw[1], 3), "-", round(ci_gw[2], 3), ")\n")

cat("P = ", format.pval(summary(model_dr)$coefficients["GW", "Pr(>|t|)"], digits = 3), "\n")

if (!require("CBPS")) install.packages("CBPS")

library(CBPS)

cbps_fit <- CBPS(Plasma ~ GW + Weight + DM + Sex + X1_Apgar + X5_Apgar +

GH + GD + NRDS + ES + Ventilator + INR + Fib + VA + PLT,

data = my_dataframe, method = "over")

my_dataframe$weights_cbps <- cbps_fit$weights

weights_cbps_trimmed <- ifelse(my_dataframe$weights_cbps < quantile(my_dataframe$weights_cbps, 0.025) |

my_dataframe$weights_cbps > quantile(my_dataframe$weights_cbps, 0.975),

NA, my_dataframe$weights_cbps)

data_cbps <- my_dataframe[!is.na(weights_cbps_trimmed), ]

design_cbps <- svydesign(ids = ~1, weights = ~weights_cbps_trimmed, data = data_cbps)

model_cbps <- svyglm(IVH ~ Plasma, design = design_cbps, family = binomial())

cat("=== comparsion of different weighting methods ===\n")

cat("GBM OR: ", round(exp(coef(model_dr)["Plasma1"]), 3), "\n")

cat("CBPS OR: ", round(exp(coef(model_cbps)["Plasma1"]), 3), "\n")

> or_cbps_point <- exp(coef(model_cbps)["Plasma1"])

> se_cbps <- summary(model_cbps)$coefficients["Plasma1", "Std. Error"]

> ci_log <- c(

+	coef(model_cbps)["Plasma1"] - 1.96 * se_cbps,

+	coef(model_cbps)["Plasma1"] + 1.96 * se_cbps

+ )

> ci_cbps <- exp(ci_log)

cat("=== CBPS model effect estimation ===\n")

> cat("CBPS OR value: ", round(or_cbps_point, 3), "\n")

> cat("95% CI: [", round(ci_cbps[1], 3), ", ", round(ci_cbps[2], 3), "]\n", sep = "")

> summary_cbps <- summary(model_cbps)

> p_value_cbps <- summary_cbps$coefficients["Plasma1", "Pr(>|t|)"]

> cat("=== CBPS model p value ===\n")

> cat("Plasma1 Pvalue: ", round(p_value_cbps, 3), "\n")

library(forestplot)

>

> forest_data <- data.frame(

+	label = c(

+	"GBM (main)",

+	"GBM (dr, adj-GW)",

+	"CBPS "

+	),

+	mean = c(1.182, 1.033, 1.260),

+	lower = c(0.787, 0.676, 0.814),

+	upper = c(1.775, 1.578, 1.948)

+ )

>

> forestplot(

+	labeltext = forest_data$label,

+	mean = forest_data$mean,

+	lower = forest_data$lower,

+	upper = forest_data$upper,

+	new_page = TRUE,

+	is.summary = c(FALSE, FALSE, FALSE),

+	xlog = FALSE,

+	zero = 1,

+	xlab = "Odds Ratio (95% CI)",

+	title = "Effect estimates for prophylactic plasma transfusion and IVH association across different weighting methods",

+	xticks = c(0.5, 1, 1.5, 2),

+	col = fpColors(

+	box = "royalblue",

+	line = "darkblue",

+	summary = "royalblue"

+	),

+	csvmx = 1,

+	align = "l", +	graph.pos = 2,

+	txt_gp = fpTxtGp(

+	label = gpar(fontsize = 12),

+	ticks = gpar(fontsize = 10),

+	xlab = gpar(fontsize = 12),

+	title = gpar(fontsize = 14, fontface = "bold")

+	)

+ )

> if (!is.factor(my_dataframe_trimmed$Plasma)) {

+	my_dataframe_trimmed$Plasma <- factor(my_dataframe_trimmed$Plasma)

+	cat("Plasma was recoded as a factor.\n")

+ }

>

> gw_mean <- mean(my_dataframe_trimmed$GW, na.rm = TRUE)

> my_dataframe_trimmed$GW_centered <- my_dataframe_trimmed$GW - gw_mean

> design_centered <- svydesign(ids = ~1, weights = ~weights_trimmed, data = my_dataframe_trimmed)

> model_interaction_corrected <- svyglm(IVH ~ Plasma * GW_centered,

+	design = design_centered,

+	family = binomial())

In eval(family$initialize) : non-integer #successes in a binomial glm!>

> summary(model_interaction_corrected)

> library(ggplot2)

>

> cat("=== data checking ===\n")

> cat("Plasmatype：", class(my_dataframe_trimmed$Plasma), "\n")

> cat("GW_centered whether existing：", "GW_centered" %in% names(my_dataframe_trimmed), "\n")

GW_centeredwhether existing： TRUE

>

> gw_mean <- mean(my_dataframe_trimmed$GW, na.rm = TRUE)

> cat("mean GA：", round(gw_mean, 1), "day\n")

> gw_seq <- seq(min(my_dataframe_trimmed$GW),

+	max(my_dataframe_trimmed$GW),

+	length.out = 100)

>

> pred_data <- expand.grid(

+	Plasma = unique(my_dataframe_trimmed$Plasma),

+	GW_centered = gw_seq - gw_mean

+ )

> pred_data$predicted_risk <- predict(model_interaction_centered,

+	newdata = pred_data,

+	type = "response")

> pred_data$GW <- pred_data$GW_centered + gw_mean

cat("\n=== Weighted study design ===\n")

design_trimmed <- svydesign(

ids = ~1,

weights = ~weights_trimmed,

data = my_dataframe_trimmed

)

cat("using 2.5% trimmed weights，sample size:", nrow(my_dataframe_trimmed), "\n")

cat("\n=== building interaction model ===\n")

model_interaction <- svyglm(

NICH ~ Plasma * GW_centered + Weight + Sex,

design = design_trimmed,

family = binomial()

)

cat("constructed model\n")

cat("\n=== the result of interaction model ===\n")

model_summary <- summary(model_interaction)

print(model_summary)

> interaction_plot <- ggplot(pred_data,

+	aes(x = GW, y = predicted_risk,

+	color = factor(Plasma),

+	linetype = factor(Plasma))) +

+	geom_line(size = 1.2) +

+	labs(title = "The effect of Plasma therapy on IVH risk is modified by gestational age.",

+	subtitle = "interaction Pvalue = 0.011",

+	x = "GA (day)",

+	y = "Predicted risk of IVH",

+	color = "treatment group",

+	linetype = "treatment group") +

+	theme_minimal(base_size = 12) +

+	scale_color_manual(values = c("0" = "blue", "1" = "red"),

+	labels = c("0" = "control group", "1" = "Plasma grouop")) +

+	scale_linetype_manual(values = c("0" = "dashed", "1" = "solid"),

+	labels = c("0" = "control group", "1" = "Plasma group")) +

+	theme(legend.position = "top")

> print(interaction_plot)

> ggsave("interaction_effect_plot.png",

+	plot = interaction_plot,

+	width = 8, height = 6, dpi = 300)

> cat("interaction_effect_plot.png\n")

library(cobalt)

> library(survey)

> cat("=== Construct an adjusted interaction model ===\n")

>

> if (!"GW_centered" %in% names(my_dataframe_trimmed)) {

+	gw_mean <- mean(my_dataframe_trimmed$GW, na.rm = TRUE)

+	my_dataframe_trimmed$GW_centered <- my_dataframe_trimmed$GW - gw_mean

+	cat(" bulided GW_centered Variable，mean GA =", round(gw_mean, 1), "天\n")

+ }

> design_centered <- svydesign(

+	ids = ~1,

+	weights = ~weights_trimmed,

+	data = my_dataframe_trimmed

+ )

> model_interaction_adj <- svyglm(

+	IVH ~ Plasma * GW_centered + Weight + Sex,

+	design = design_centered,

+	family = binomial()

+ )

> cat("\nresult：\n")

> print(summary(model_interaction_adj))

> interaction_p <- summary(model_interaction_adj)$coefficients["Plasma1:GW_centered", "Pr(>|t|)"]

> cat("interaction Plasma1:GW_centered 的Pvalue =", round(interaction_p, 4), "\n")

**library(forestplot)**

**library(grid)**

**create_forest_labels <- function(forest_data) {**

**n_total_subgroups <- sum(forest_data$N_Total[-1])**

**weight_percent <- c("100%",**

**sprintf("%.1f%%",**

**forest_data$N_Total[-1]/n_total_subgroups*100))**

**labels <- cbind(**

**c("subgroups", as.character(forest_data$Subgroup)),**

**c("sample size\n(weighting%)",**

**paste0("n=", forest_data$N_Total, "\n",**

**weight_percent)),**

**c("treatment group/control group\n(IVH)",**

**paste0(forest_data$IVH_Treat, "/", forest_data$N_Treat,**

**" vs\n",**

**forest_data$IVH_Control, "/", forest_data$N_Control)),**

**c("OR (95% CI)",**

**sprintf("%.2f (%.2f-%.2f)",**

**forest_data$OR,**

**forest_data$CI_Lower,**

**forest_data$CI_Upper)),**

**c("Pvalue",**

**ifelse(forest_data$P_Value < 0.001, "<0.001",**

**sprintf("%.3f", forest_data$P_Value)))**

**)**

**return(labels)**

**}**

**cat("preparing data...\n")**

**if (!exists("forest_data")) {**

**forest_data <- data.frame(**

**Subgroup = c("overall", "low-GA group (<critical point)", "high-GA group (≥critical point)"),**

**N_Total = c(710, 350, 360),**

**N_Treat = c(425, 200, 225),**

**N_Control = c(285, 150, 135),**

**IVH_Treat = c(42, 25, 17),**

**IVH_Control = c(30, 12, 18),**

**OR = c(1.02, 1.25, 0.85),**

**CI_Lower = c(0.67, 0.85, 0.55),**

**CI_Upper = c(1.56, 1.85, 1.32),**

**P_Value = c(0.910, 0.230, 0.470)**

**)**

**}**

**cat("	rows:", nrow(forest_data), "\n")**

**cat("2. buliding labels...\n")**

**label_text <- create_forest_labels(forest_data)**

**cat(" building labe，dimension:", dim(label_text)[1], "row x", dim(label_text)[2], "column\n")**

**cat("3. preparing data...\n")**

**mean_values <- c(NA, forest_data$OR)**

**lower_values <- c(NA, forest_data$CI_Lower)**

**upper_values <- c(NA, forest_data$CI_Upper)**

**box_colors <- c("black",**

**ifelse(forest_data$P_Value[-1] < 0.05, "red", "blue"))**

**line_colors <- c("black",**

**ifelse(forest_data$P_Value[-1] < 0.05, "darkred", "darkblue"))**

**cat("4. building forest plot...\n")**

**png("subgroup_forestplot_corrected.png", width = 1000, height = 600, res = 150)**

**forestplot(labeltext = label_text,**

**mean = mean_values,**

**lower = lower_values,**

**upper = upper_values,**

**graph.pos = 3,**

**is.summary = c(TRUE, rep(FALSE, nrow(forest_data))),**

**clip = c(0.2, 5),**

**xlog = TRUE,**

**col = fpColors(box = box_colors,**

**line = line_colors,**

**summary = "black"),**

**txt_gp = fpTxtGp(label = gpar(cex = 0.8),**

**ticks = gpar(cex = 0.7),**

**xlab = gpar(cex = 0.9)),**

**grid = structure(c(0.5, 1, 2, 5),**

**gp = gpar(lty = 2, col = "gray")),**

**xlab = " (OR) 与 95% CI",**

**title = paste0("analysis of subgroup\n",**

**"based on the critical point: ",**

**ifelse(exists("gw_critical"),**

**paste0(round(gw_critical/7, 2), "week"),**

**"undefined")),**

**boxsize = 0.2,**

**line.margin = 0.1,**

**colgap = unit(5, "mm"),**

**graphwidth = unit(4, "in"))**

**grid.text("red: P < 0.05 (significant) | blue: P ≥ 0.05 (not significant)",**

**x = 0.5, y = 0.02,**

**just = "center",**

**gp = gpar(cex = 0.8, col = "gray40"))**

**dev.off()**

**cat("✅ subgroup_forestplot_corrected.png\n")**

**cat("C:", getwd(), "/subgroup_forestplot_corrected.png\n")**

**if (!require("ggplot2")) install.packages("ggplot2")**

**if (!require("dplyr")) install.packages("dplyr")**

**library(ggplot2)**

**library(dplyr)**

**forest_ggplot_data <- forest_data**

**forest_ggplot_data$Subgroup <- factor(forest_ggplot_data$Subgroup,**

**levels = rev(forest_ggplot_data$Subgroup))**

**forest_ggplot_data$Significance <- ifelse(forest_ggplot_data$P_Value < 0.05,**

**"significant (P < 0.05)", "not significant (P ≥ 0.05)")**

**forest_ggplot_data$x_text <- forest_ggplot_data$OR**

**forest_ggplot_data$x_text_adj <- ifelse(forest_ggplot_data$OR < 1,**

**forest_ggplot_data$CI_Lower * 0.7,**

**forest_ggplot_data$CI_Upper * 1.3)**

**ggplot_forest <- ggplot(forest_ggplot_data, aes(x = OR, y = Subgroup)) +**

**geom_vline(xintercept = 1, linetype = "dashed", color = "red", linewidth = 0.8) +**

**geom_errorbarh(aes(xmin = CI_Lower, xmax = CI_Upper, color = Significance),**

**height = 0.2, linewidth = 1.2) +**

**geom_point(aes(fill = Significance, size = N_Total), shape = 22, stroke = 1) +**

**geom_text(aes(x = x_text_adj,**

**label = sprintf("OR=%.2f\n(%.2f-%.2f)", OR, CI_Lower, CI_Upper)),**

**size = 3.5, vjust = 0.5, hjust = 0.5, color = "black") +**

**geom_text(aes(x = 0.15,**

**label = paste0("n=", N_Total, "\n",**

**IVH_Treat, "/", N_Treat, " vs ",**

**IVH_Control, "/", N_Control)),**

**size = 3.2, hjust = 0, color = "gray30") +**

**scale_fill_manual(values = c("significant (P < 0.05)" = "firebrick",**

**"no significant (P ≥ 0.05)" = "steelblue")) +**

**scale_color_manual(values = c("significant (P < 0.05)" = "firebrick3",**

**"no significant (P ≥ 0.05)" = "steelblue3")) +**

**scale_size_continuous(range = c(4, 10),**

**breaks = c(100, 300, 500, 700),**

**name = "sample size") +**

**scale_x_log10(breaks = c(0.2, 0.5, 1, 2, 5),**

**labels = c("0.2", "0.5", "1.0", "2.0", "5.0"),**

**limits = c(0.1, 10)) +**

**labs(**

**title = "analysis of subgroup results",**

**subtitle = ifelse(exists("gw_critical"),**

**paste0("based on the critical point: ", round(gw_critical/7, 2), "week",**

**ifelse(exists("p_interaction"),**

**paste0(" | interaction P = ",**

**format.pval(p_interaction, digits = 3)),**

**"")),**

**"result of subgroup"),**

**x = "(OR) 与 95% CI",**

**y = "",**

**fill = "significance",**

**color = "CI"**

**) +**

**theme_minimal(base_size = 12) +**

**theme(**

**plot.title = element_text(face = "bold", hjust = 0.5, size = 16,**

**margin = margin(b = 10)),**

**plot.subtitle = element_text(hjust = 0.5, color = "gray40", size = 12,**

**margin = margin(b = 15)),**

**axis.title.x = element_text(face = "bold", size = 12,**

**margin = margin(t = 10)),**

**axis.text.y = element_text(face = "bold", size = 11, color = "black"),**

**axis.text.x = element_text(size = 10),**

**panel.grid.major.y = element_line(color = "gray90", linewidth = 0.5),**

**panel.grid.minor.y = element_blank(),**

**panel.grid.major.x = element_line(color = "gray90", linewidth = 0.5),**

**panel.grid.minor.x = element_blank(),**

**legend.position = "bottom",**

**legend.box = "vertical",**

**legend.box.just = "left",**

**legend.margin = margin(t = 5, b = 5),**

**legend.title = element_text(face = "bold", size = 10),**

**legend.text = element_text(size = 9),**

**plot.margin = margin(20, 20, 20, 20)**

**) +**

**facet_grid(rows = vars(ifelse(grepl("overall", Subgroup), "overall", "subgroup")),**

**scales = "free_y", space = "free_y") +**

**theme(strip.text = element_text(face = "bold", size = 11),**

**strip.background = element_rect(fill = "gray95", color = NA))**

**print(ggplot_forest)**

**ggplot_pdf_file <- "subgroup_forestplot_ggplot.pdf"**

**ggsave(ggplot_pdf_file, plot = ggplot_forest,**

**width = 12, height = 8, device = "pdf", dpi = 300)**

**cat("\n✅ ggplot2saved: ", ggplot_pdf_file, "\n")**

cat("\n=== sensitivity analysis ===\n")

model_unweighted <- glm(

as.formula(paste(outcome_var, "~", treatment_var)),

data = my_dataframe_trimmed,

family = binomial()

)

or_unweighted <- exp(coef(model_unweighted)[paste0(treatment_var, "1")])

ci_unweighted <- exp(confint(model_unweighted)[paste0(treatment_var, "1"), ])

cat("Results of the unweighted model：\n")

cat("ORvalue：", round(or_unweighted, 3), "\n")

cat("95% CI：[", round(ci_unweighted[1], 3), ", ", round(ci_unweighted[2], 3), "]\n")

> cat("\n=== Sensitivity analysis using different trimming thresholds ===\n")

> trim_levels <- c(0.01, 0.025, 0.05)

> results_sensitivity <- data.frame()

> for (level in trim_levels) {

+	lower <- quantile(weight_gbm$weights, level/2, na.rm = TRUE)

+	upper <- quantile(weight_gbm$weights, 1 - level/2, na.rm = TRUE)

+	data_temp <- my_dataframe

+	data_temp$weights_trimmed <- weight_gbm$weights

+	data_temp$weights_trimmed[data_temp$weights_trimmed < lower | data_temp$weights_trimmed > upper] <- NA

+	data_temp <- data_temp[!is.na(data_temp$weights_trimmed), ]

+	if (nrow(data_temp) < 10) {

+	cat(paste0("warning", level*100, "%sample size was too small，running\n"))

+	next

+	}

+	design_temp <- svydesign(ids = ~1, weights = ~weights_trimmed, data = data_temp)

+	model_temp <- svyglm(as.formula(paste(outcome_var, "~", treatment_var)),

+	design = design_temp, family = binomial())

+	or_temp <- exp(coef(model_temp)[paste0(treatment_var, "1")])

+	ci_temp <- exp(confint(model_temp)[paste0(treatment_var, "1"), ])

+	p_temp <- summary(model_temp)$coefficients[paste0(treatment_var, "1"), "Pr(>|t|)"]

+	results_sensitivity <- rbind(results_sensitivity,

+	data.frame(

+	Trim_Level = paste0(level*100, "%"),

+	Sample_Size = nrow(data_temp),

+	OR = round(or_temp, 3),

+	CI_Lower = round(ci_temp[1], 3),

+	CI_Upper = round(ci_temp[2], 3),

+	P_Value = round(p_temp, 3)

+	))

+ }

> print(results_sensitivity)

cat("\n=== saving result ===\n")

write.csv(my_dataframe_trimmed, "data_gbm_weighted_trimmed.csv", row.names = FALSE)

cat("saved result：data_gbm_weighted_trimmed.csv\n")

save(weight_gbm, file = "gbm_weight_object.RData")

cat("saved result：gbm_weight_object.RData\n")

save(model_weighted, balance_result, file = "analysis_results.RData")

cat("saved result：analysis_results.RData\n")

cat("\n✅ GBMweighting ended！\n")

library(survey)

beta <- coef(model_dr)["Plasma1"]

se <- summary(model_dr)$coefficients["Plasma1", "Std. Error"]

or_est <- exp(beta)

ci_lower <- exp(beta - 1.96 * se)

ci_upper <- exp(beta + 1.96 * se)

cat("=== result of model ===\n")

cat("beta: ", round(beta, 4), "\n")

cat("se: ", round(se, 4), "\n")

cat("OR: ", round(or_est, 4), "\n")

cat("95% CI: [", round(ci_lower, 4), ", ", round(ci_upper, 4), "]\n")

calculate_evalue <- function(rr) {

if (rr >= 1) {

return(rr + sqrt(rr * (rr - 1)))

} else {

return((1/rr) + sqrt((1/rr) * ((1/rr) - 1)))

}

}

evalue_point <- calculate_evalue(or_est)

dist_to_null <- c(abs(ci_lower - 1), abs(ci_upper - 1))

if (dist_to_null[1] < dist_to_null[2]) {

nearest_or <- ci_lower

} else {

nearest_or <- ci_upper

}

evalue_bound <- calculate_evalue(nearest_or)

cat("\n=== Evalue ===\n")

cat("based on point estimation OR = ", round(or_est, 3), " E: ", round(evalue_point, 3), "\n")

cat("Based on the boundary closest to non-inferiority OR = ", round(nearest_or, 3), " E: ", round(evalue_bound, 3), "\n")

cat("\n=== sensitivity analysis ===\n")

cat("E = ", round(evalue_point, 3), " show：\n")

cat("unmeasured U，=：\n")

cat("1. The strength of association between U and treatment (Plasma) ≥ ", round(evalue_point, 3), " fold\n")

cat("2. The strength of association between U and outcome (IVH) ≥ ", round(evalue_point, 3), " fold\n")

cat("3. to fully explain the observed effect of OR = ", round(or_est, 3), "\n\n")

gw_or <- exp(coef(model_dr)["GW"])

gw_strength <- 1/gw_or # Convert to risk direction

cat("=== Comparison with the known strongest confounder (GW) ===\n")

cat("Effect of GW: OR = ", round(gw_or, 3), " (per 1-day increase in gestational age)\n")

cat("Strength of association for GW: ", round(gw_strength, 3), " fold\n")

cat("Ratio of E-value to GW strength: ", round(evalue_point / gw_strength, 3), "\n")

par(mfrow = c(1, 2))

strengths <- c(evalue_point, gw_strength)

names(strengths) <- c("unmeasured (E value)", "(GW)")

barplot(strengths,

col = c("lightblue", "lightgreen"),

ylab = "threshold",

main = "Comparison with the known strongest confounder (GW)",

ylim = c(0, max(strengths) * 1.3))

gammas <- seq(1, 2, by = 0.1)

p_values <- sapply(gammas, function(g) {

adjusted_or <- or_est / g

adjusted_se <- se * 1.1

z <- log(adjusted_or) / adjusted_se

2 * pnorm(-abs(z))

})

plot(gammas, p_values, type = "b",

xlab = "Gamma",

ylab = "adjusted Pvalue",

main = "the effect of unmeasured confounder on Pvalue",

ylim = c(0, 1))

abline(h = 0.05, col = "red", lty = 2)

abline(v = evalue_point, col = "blue", lty = 2)

legend("topright",

legend = c("P=0.05 threshold", paste("Evalue=", round(evalue_point,2))),

col = c("red", "blue"), lty = 2)

par(mfrow = c(1, 1))

if (!exists("design")) {

design <- svydesign(ids = ~1, weights = ~weights_trimmed,

data = my_dataframe_trimmed)

}

model_nc <- svyglm(

Weight ~ Plasma, design = design

)

cat("=== negative analysis ===\n")

cat("Hypothesis: No significant effect of Plasma on birth weight\n")

cat("result：\n")

print(summary(model_nc))

if (summary(model_nc)$coefficients["Plasma1", "Pr(>|t|)"] < 0.05) {

cat("warning\n")

} else {

cat("✅ no significance\n")

}

cat("=== negative analysis after adjusting Gw ===\n")

model_nc_adj <- svyglm(

Weight ~ Plasma + GW,

design = svydesign(ids = ~1, weights = ~weights_trimmed,

data = my_dataframe_trimmed)

)

cat("model after adjusting：\n")

print(summary(model_nc_adj))

p_adj <- summary(model_nc_adj)$coefficients["Plasma1", "Pr(>|t|)"]

cat("\n adjusting GW P value ：", p_adj, "\n")

par(mfrow = c(1, 2))

boxplot(Weight ~ Plasma, data = my_dataframe_trimmed,

main = "distribution of GW",

xlab = "treatment group", ylab = "BW (g)",

col = c("lightblue", "lightpink"))

plot(density(my_dataframe_trimmed$Weight[my_dataframe_trimmed$Plasma == 0], na.rm = TRUE),

main = "distribution of BW", col = "blue", lwd = 2,

xlab = "BW (g)", ylab = "density")

lines(density(my_dataframe_trimmed$Weight[my_dataframe_trimmed$Plasma == 1], na.rm = TRUE),

col = "red", lwd = 2)

legend("topright", legend = c("control group", "treatment group"),

col = c("blue", "red"), lwd = 2)

par(mfrow = c(1, 1))

library(ggplot2)

if ("weights_gbm" %in% names(my_dataframe)) {

plot_data <- data.frame(

weight = my_dataframe$weights_gbm,

Plasma = factor(my_dataframe$Plasma, levels = c(0, 1),

labels = c("control group", "treatment group"))

)

print(simple_plot)

ggsave("simple_weight_overlap.png", width = 8, height = 6, dpi = 300)

cat("saved\n")

} else {

cat("no weighting column\n")

}

if (!require("ggplot2")) {

install.packages("ggplot2")

}

library(ggplot2)

if (!require("dplyr")) {

install.packages("dplyr")

}

library(dplyr)

cat("=== preparing data ===\n")

if (exists("weight_gbm_optimized")) {

cat(" weight_gbm_optimized\n")

if (!is.null(weight_gbm_optimized$ps)) {

propensity_scores <- weight_gbm_optimized$ps

cat(" $ps extract PS score\n")

} else if (!is.null(weight_gbm_optimized$distance)) {

propensity_scores <- weight_gbm_optimized$distance

cat("从 $distance extract PS score\n")

} else {

cat("warning\n")

if ("ps" %in% names(my_dataframe)) {

propensity_scores <- my_dataframe$ps

cat("my_dataframe$ps extract ps score\n")

} else {

cat("calculate simple...\n")

ps_model <- glm(Plasma ~ GW, data = my_dataframe, family = binomial())

propensity_scores <- predict(ps_model, type = "response")

}

}

} else {

cat("warning 'weight_gbm_optimized'\n")

cat("calculate simple PS score...\n")

ps_model <- glm(Plasma ~ GW, data = my_dataframe, family = binomial())

propensity_scores <- predict(ps_model, type = "response")

}

cat("ps score:", length(propensity_scores), "\n")

if (length(propensity_scores) == nrow(my_dataframe)) {

plasma_var <- my_dataframe$Plasma

cat("initial data:", nrow(my_dataframe), "\n")

} else if (exists("my_dataframe_trimmed") &&

length(propensity_scores) == nrow(my_dataframe_trimmed)) {

plasma_var <- my_dataframe_trimmed$Plasma

cat("trimmed sample:", nrow(my_dataframe_trimmed), "\n")

} else {

min_len <- min(length(propensity_scores), nrow(my_dataframe))

propensity_scores <- propensity_scores[1:min_len]

plasma_var <- my_dataframe$Plasma[1:min_len]

cat(":", min_len, "\n")

}

plot_data <- data.frame(

ps = propensity_scores,

Plasma = factor(plasma_var, levels = c(0, 1), labels = c("control group", "treatment group"))

)

cat("rows:", nrow(plot_data), "\n")

library(overlapping)

if (sum(plot_data$Plasma == "control group") > 1 && sum(plot_data$Plasma == "treatment group") > 1) {

ps_list <- list(

control = plot_data$ps[plot_data$Plasma == "control group"],

treat = plot_data$ps[plot_data$Plasma == "treatment group"]

)

overlap_result <- overlap(ps_list)

cat("overlapping:", round(overlap_result$OV, 3), "\n")

}

cat("\n=== overlapping plot ===\n")

overlap_plot <- ggplot(plot_data, aes(x = ps, fill = Plasma)) +

geom_density(alpha = 0.6, adjust = 1.2) +

geom_vline(xintercept = 0.5, linetype = "dashed", color = "gray40", size = 0.8) +

scale_fill_manual(values = c("control group" = "#377eb8", "treatment group" = "#e41a1c")) +

labs(

title = "overlapping plot",

subtitle = "Check common support domain",

x = "PS (treatment probability)",

y = "density",

fill = "treatment group"

) +

theme_minimal(base_size = 12) +

theme(

plot.title = element_text(hjust = 0.5, face = "bold"),

plot.subtitle = element_text(hjust = 0.5, color = "gray40"),

legend.position = "top"

) +

xlim(0, 1)

print(overlap_plot)

ggsave("propensity_score_overlap_corrected.png",

plot = overlap_plot,

width = 8, height = 6, dpi = 300)

2. Causal forest

if (!require("pacman")) install.packages("pacman")

pacman::p_load(tidyverse, grf, rpart, rpart.plot, ggplot2, gridExtra, scales)

setwd("D:/yqy/new")

data_raw <- read.csv("IVH.csv", header = TRUE)

colnames(data_raw) <- make.names(colnames(data_raw))

y_temp <- as.character(data_raw$IVH)

if(!all(unique(y_temp) %in% c("No", "Yes"))) {

tbl <- table(y_temp); minority <- names(tbl)[which.min(tbl)]

y_temp <- ifelse(y_temp == minority, "Yes", "No")

}

data_raw$Y_binary <- ifelse(tolower(y_temp) %in% c("yes", "1", "true"), 1, 0)

p_temp <- as.character(data_raw$Plasma)

treat_label <- unique(p_temp)[grepl("Yes|1|Trans|True", unique(p_temp), ignore.case = TRUE)][1]

if(is.na(treat_label)) { tbl_p <- table(p_temp); treat_label <- names(tbl_p)[which.max(tbl_p)] }

data_raw$W_binary <- ifelse(data_raw$Plasma == treat_label, 1, 0)

baseline_vars <- c("GW", "Weight", "DM", "Sex", "X1_Apgar", "X5_Apgar",

"GH", "GD", "NRDS", "ES", "Ventilator", "APTT", "INR",

"Fib", "VA", "PLT")

baseline_vars <- baseline_vars[baseline_vars %in% names(data_raw)]

df_cf <- data_raw %>% select(all_of(c("Y_binary", "W_binary", baseline_vars))) %>% na.omit()

Y <- df_cf$Y_binary

W <- df_cf$W_binary

X_formula <- as.formula(paste("~", paste(baseline_vars, collapse = "+"), "- 1"))

X <- model.matrix(X_formula, data = df_cf)

set.seed(123)

cf_model <- causal_forest(X, Y, W, num.trees = 3000, seed = 123)

tau_hat <- predict(cf_model, estimate.variance = TRUE)

df_cf$ITE <- tau_hat$predictions

df_cf$ITE_Lower <- df_cf$ITE - 1.96 * sqrt(tau_hat$variance.estimates)

df_cf$ITE_Upper <- df_cf$ITE + 1.96 * sqrt(tau_hat$variance.estimates)

df_cf$Effect_Type <- case_when(

df_cf$ITE_Lower > 0 ~ "Harmful (Red)",

df_cf$ITE_Upper < 0 ~ "Beneficial (Blue)",

TRUE ~ "Neutral (Grey)"

)

ate <- average_treatment_effect(cf_model, target.sample = "all")

ate_df <- data.frame(

Label = "Global Average Effect",

Estimate = ate["estimate"],

Lower = ate["estimate"] - 1.96 * ate["std.err"],

Upper = ate["estimate"] + 1.96 * ate["std.err"]

)

p1_ate <- ggplot(ate_df, aes(x = Label, y = Estimate)) +

geom_hline(yintercept = 0, linetype = "dashed", color = "gray50", linewidth = 1) +

geom_errorbar(aes(ymin = Lower, ymax = Upper), width = 0.2, linewidth = 1, color = "#2C3E50") +

geom_point(size = 5, color = "#E74C3C") +

geom_text(aes(label = paste0("ATE: ", round(Estimate*100, 2), "%\n(P=",

round(2*(1-pnorm(abs(ate["estimate"]/ate["std.err"]))), 3), ")")),

hjust = -0.3, vjust = 0.5, size = 5) +

coord_flip() +

labs(title = "Figure 1: Global Average Treatment Effect (ATE)",

subtitle = "The 95% CI crosses zero, indicating no statistically significant global effect.",

y = "Risk Difference (Treatment - Control)", x = "") +

theme_bw() +

theme(axis.text.y = element_text(size = 12, face = "bold"),

plot.title = element_text(face = "bold"))

ggsave("Fig1_Global_ATE.pdf", p1_ate, width = 8, height = 4)

p2_hist <- ggplot(df_cf, aes(x = ITE)) +

geom_histogram(aes(y = after_stat(density)), bins = 30, fill = "#3498DB", alpha = 0.6, color = "white") +

geom_density(color = "#2980B9", linewidth = 1.2) +

geom_vline(xintercept = 0, linetype = "dashed", color = "red", linewidth = 1) +

labs(title = "Figure 2: Distribution of Individual Treatment Effects",

subtitle = "Most patients cluster around 0 (no effect), with a slight skew.",

x = "Estimated Risk Difference (Individual Level)", y = "Density") +

theme_bw()

ggsave("Fig2_ITE_Distribution.pdf", p2_hist, width = 8, height = 6)

plot_data <- df_cf %>% arrange(ITE) %>% mutate(Rank = 1:n())

p3_waterfall <- ggplot(plot_data, aes(x = Rank, y = ITE, fill = Effect_Type, color = Effect_Type)) +

geom_bar(stat = "identity", width = 1) +

scale_fill_manual(values = c("Harmful (Red)" = "#E74C3C",

"Beneficial (Blue)" = "#3498DB",

"Neutral (Grey)" = "grey85")) +

scale_color_manual(values = c("Harmful (Red)" = "#E74C3C",

"Beneficial (Blue)" = "#3498DB",

"Neutral (Grey)" = "grey85")) +

labs(title = "Figure 3: Heterogeneity Waterfall Plot",

subtitle = "Sorted by estimated treatment effect. Colored bars indicate significant effects (95% CI excludes 0).",

y = "Risk Difference (Plasma - No Plasma)", x = "Patients") +

theme_bw() + theme(legend.position = "bottom")

ggsave("Fig3_Waterfall.pdf", p3_waterfall, width = 9, height = 6)

var_imp <- variable_importance(cf_model)

var_imp_df <- data.frame(Variable = colnames(X), Importance = var_imp[,1]) %>%

arrange(desc(Importance)) %>%

head(10)

p4_imp <- ggplot(var_imp_df, aes(x = reorder(Variable, Importance), y = Importance)) +

geom_col(fill = "#2ECC71", width = 0.7) +

coord_flip() +

labs(title = "Figure 4: Top 10 Drivers of Heterogeneity",

subtitle = "Which baseline characteristics modify the treatment effect most?",

x = "", y = "Relative Importance") +

theme_bw() +

theme(axis.text.y = element_text(size = 11))

ggsave("Fig4_Variable_Importance.pdf", p4_imp, width = 7, height = 6)

tree_data <- data.frame(X, ITE = df_cf$ITE)

fit_tree <- rpart(ITE ~ ., data = tree_data,

control = rpart.control(cp = 0.005, maxdepth = 4))

pdf("Fig5_Subgroup_Tree.pdf", width = 11, height = 8)

rpart.plot(fit_tree, type = 4, extra = 101, under = TRUE, digits = 3,

box.palette = "RdBu", shadow.col = "gray", nn = TRUE,

main = "Figure 5: Decision Tree for Identifying High-Risk Groups",

tweak = 1.1)

dev.off()

ate_est <- ate["estimate"]

ate_se <- ate["std.err"]

ci_lower <- ate_est - 1.96 * ate_se

ci_upper <- ate_est + 1.96 * ate_se

p_val <- 2 * (1 - pnorm(abs(ate_est / ate_se)))

if (!require("tableone")) {

install.packages("tableone")

library(tableone)

}

df_table1 <- df_cf

df_table1$Group <- factor(ifelse(df_table1$W_binary == 1, "Plasma (Treat)", "No Plasma (Control)"))

df_table1$IVH_Outcome <- factor(ifelse(df_table1$Y_binary == 1, "Yes", "No"))

categorical_vars <- c("DM", "Sex", "GH", "GD", "NRDS", "ES", "Ventilator", "IVH_Outcome")

categorical_vars <- categorical_vars[categorical_vars %in% colnames(df_table1)]

vars_to_include <- c(baseline_vars, "IVH_Outcome")

tab1 <- CreateTableOne(

vars = vars_to_include,

strata = "Group",

data = df_table1,

factorVars = categorical_vars,

addOverall = TRUE

)

print(tab1, showAllLevels = TRUE, quote = FALSE, noSpaces = TRUE, formatOptions = list(big.mark = ","))

tab1_mat <- print(tab1, showAllLevels = TRUE, quote = FALSE, noSpaces = TRUE, printToggle = FALSE)

write.csv(tab1_mat, file = "Table1_Baseline_Characteristics.csv", row.names = TRUE)
